# Periostin‐ and podoplanin‐positive cancer‐associated fibroblast subtypes cooperate to shape the inflamed tumor microenvironment in aggressive pancreatic adenocarcinoma

**DOI:** 10.1002/path.6011

**Published:** 2022-10-21

**Authors:** Cindy Neuzillet, Rémy Nicolle, Jérôme Raffenne, Annemilaï Tijeras‐Raballand, Alexia Brunel, Lucile Astorgues‐Xerri, Sophie Vacher, Floriane Arbateraz, Marjorie Fanjul, Marc Hilmi, Rémi Samain, Christophe Klein, Aurélie Perraud, Vinciane Rebours, Muriel Mathonnet, Ivan Bièche, Hemant Kocher, Jérôme Cros, Corinne Bousquet

**Affiliations:** ^1^ Department of Medical Oncology, Institut Curie Université Versailles Saint‐Quentin, Paris Saclay Saint‐Cloud France; ^2^ UMR144, Institut Curie Paris France; ^3^ INSERM U1149, Centre de Recherche sur l'Inflammation Paris France; ^4^ INSERM UMR‐1037, Cancer Research Center of Toulouse (CRCT), Team ‘labellisée Ligue Contre le Cancer’ University of Toulouse Toulouse France; ^5^ OncoMEGA Lamorlaye France; ^6^ Department of Genetics Institut Curie, PSL Research University Paris France; ^7^ Centre d'Histologie Imagerie et Cytométrie (CHIC), U1138 Centre de Recherche des Cordeliers (CRC) Paris France; ^8^ Department of Digestive Surgery University Hospital of Limoges Limoges France; ^9^ INSERM UMLR‐1308 University of Limoges Limoges France; ^10^ Department of Pancreatology Beaujon Hospital (APHP) Clichy‐La‐Garenne France; ^11^ Centre for Tumour Biology, Barts Cancer Institute ‐ a CR‐UK Centre of Excellence Queen Mary University of London London UK; ^12^ Department of Pathology, Beaujon Hospital (APHP) Université de Paris Paris France

**Keywords:** heterogeneity, stroma, transcriptomic classification, immune cells, immunohistochemistry, RNAseq, prognosis, macrophage, dendritic cell

## Abstract

Cancer‐associated fibroblasts (CAFs) are orchestrators of the pancreatic ductal adenocarcinoma (PDAC) microenvironment. Previously we described four CAF subtypes with specific molecular and functional features. Here, we have refined our CAF subtype signatures using RNAseq and immunostaining with the goal of defining bioinformatically the phenotypic stromal and tumor epithelial states associated with CAF diversity. We used primary CAF cultures grown from patient PDAC tumors, human data sets (in‐house and public, including single‐cell analyses), genetically engineered mouse PDAC tissues, and patient‐derived xenografts (PDX) grown in mice. We found that CAF subtype RNAseq signatures correlated with immunostaining. Tumors rich in periostin‐positive CAFs were significantly associated with shorter overall survival of patients. Periostin‐positive CAFs were characterized by high proliferation and protein synthesis rates and low α‐smooth muscle actin expression and were found in peri‐/pre‐tumoral areas. They were associated with highly cellular tumors and with macrophage infiltrates. Podoplanin‐positive CAFs were associated with immune‐related signatures and recruitment of dendritic cells. Importantly, we showed that the combination of periostin‐positive CAFs and podoplanin‐positive CAFs was associated with specific tumor microenvironment features in terms of stromal abundance and immune cell infiltrates. Podoplanin‐positive CAFs identified an inflammatory CAF (iCAF)‐like subset, whereas periostin‐positive CAFs were not correlated with the published myofibroblastic CAF (myCAF)/iCAF classification. Taken together, these results suggest that a periostin‐positive CAF is an early, activated CAF, associated with aggressive tumors, whereas a podoplanin‐positive CAF is associated with an immune‐related phenotype. These two subpopulations cooperate to define specific tumor microenvironment and patient prognosis and are of putative interest for future therapeutic stratification of patients. © 2022 The Authors. *The Journal of Pathology* published by John Wiley & Sons Ltd on behalf of The Pathological Society of Great Britain and Ireland.

## Introduction

Pancreatic ductal adenocarcinoma (PDAC) will become the second leading cause of cancer death by 2030 [1]. PDACs are resistant to conventional therapies and display an unfavorable prognosis, with a 5‐year overall survival (OS) rate of less than 10% [2].

PDACs are characterized by a complex, poorly vascularized microenvironment also called stroma, made up of an excess of extracellular matrix (ECM) and different cell types, mainly cancer‐associated fibroblasts (CAFs) and protumoral, inflammatory immune cells including M2 macrophages, whereas antitumoral CD8‐positive T cells are excluded from the tumors [3, 4, 5, 6]. These biological features of PDAC make it challenging for anticancer therapies to succeed.

CAFs orchestrate the PDAC microenvironment by producing the ECM, creating a hypoxic environment through the generation of a high interstitial pressure, and interacting with tumor and other stromal cells [6]. These interactions promote tumor growth, invasion, metastasis, and resistance to therapy [3]. A major source of CAFs in PDACs are pancreatic stellate cells (PSCs), which are resident mesenchymal cells of the pancreas that in their quiescent state store vitamin‐A‐containing lipid droplets [7]. Upon activation, PSCs lose this storage function, express α‐smooth muscle actin (αSMA), and secrete ECM proteins, remodeling enzymes, and growth factors [7]. In recent years, evidence has accumulated supporting the heterogeneity of CAFs in PDAC [8, 9, 10], with coexisting putative pro‐ and antitumoral subpopulations (e.g. inflammatory CAFs [iCAF] and myofibroblastic CAFs [myCAFs], respectively) [11, 12, 13, 14, 15], which may account for the paradoxical effects of stromal depletion in these tumors [16, 17, 18, 19, 20, 21, 22, 23]. Understanding such diversity in CAF phenotypes conditions the efficiency of future antistroma therapies.

In a previous report [10], using primary cultures of human CAFs from resected PDAC, we described four CAF subtypes (A to D) with specific molecular and functional features. Based on transcriptomic analysis (using NanoString technology) and immunohistochemistry (IHC), we identified periostin (POSTN), myosin‐11 (MYH11), and podoplanin (PDPN) as markers of subtypes A, B, and C, respectively. POSTN‐positive CAFs (subtype A) were the most frequently present and were associated with poor prognosis in patients with resected PDAC.

In this study, we further refined our CAF subtype signatures using IHC and RNAseq with the goal of defining bioinformatically phenotypic stromal and tumor epithelial states associated with such CAF diversity in a variety of models: (1) a large panel of primary CAF cultures grown from patient PDAC tissue, (2) resected‐PDAC patient data sets (public and in‐house) and genetically engineered KPC mouse tissues, and (3) PDAC patient‐derived xenografts (PDX) grown in mice (a hybrid model where RNA sequences from human tumor epithelial and murine stromal cells can be distinguished) [24]. Here we show that CAF classified with the POSTN multigene RNAseq signature (subtype A) is an early, activated CAF associated with aggressive tumors, whereas CAF associated with the PDPN multigene RNAseq signature (subtype C) is associated with an immune‐related phenotype. Importantly, we found that the combination of both POSTN‐positive CAFs and PDPN‐positive CAFs was associated with specific features of the microenvironment (in terms of stromal abundance and immune cell infiltrates) and prognosis, which suggests cooperation between CAF subpopulations in PDAC.

## Materials and methods

### Patient consent and ethical approval

Ethics approval was obtained for the use of patient tumor samples. German contribution (human primary cultures): Ethics Committee of the Faculty of Medicine of the Technical University of Munich, number 1926/07; first approved 30 October 2007. Australian contribution (human primary cultures): institutional ethics approval number HREC11189/SESIAHS 00/088. French contribution (formalin‐fixed and paraffin‐embedded [FFPE] tumor samples): Beaujon biobank registration number BB‐0033‐00078. UK contribution (PSCs) UK Human Tissue Bank; Trent MREC, 05/MRE04/82. All participants gave informed consent before taking part in the study.

### Primary CAFs

Two independent sets of primary CAF cultures (Set 1, *n* = 16, and Set 2, *n* = 23) were isolated using the previously described outgrowth method (M. Apte's and M. Erkan's groups) [25]. All experiments for functional and molecular characterization of CAFs were performed on a single passage for each CAF culture. All care was taken to minimize the effect of cell growth in artificial conditions using early passages. CAF cell counting was performed using the Malassez cell counting method [26].

### Genetically engineered spontaneous mouse model of PDAC (KPC model)

KPC (*Pdx‐1‐Cre; LSL‐KrasG12D/+; LSL‐Trp53R172H/+*) mice were used [27]. Tumor development was detected by weekly ultrasound monitoring (Vevo 2,100; Fujifilm VisualSonics, Amsterdam, The Netherlands, or Aixplorer; SuperSonic Imagine, Aix‐en‐Provence, France). Mice were euthanized when the tumor became visible (around 100 mm^3^). After sacrifice, the pancreas was removed and formalin‐fixed and paraffin‐embedded (FFPE) for IHC.

Mice were kept under specific pathogen‐free conditions at the Cancer Research Center of Toulouse animal facility (CREFRE). All experiments were in compliance with institutional guidelines and European animal protection law and approved by the responsible government agency (Agreement 201612011806414).

### Beaujon Hospital patient cohort

Fifty patients were selected from a retrospective cohort of patients with localized (nonmetastatic) PDAC who had undergone complete surgical resection between December 2011 and January 2014 at Beaujon University Hospital (Clichy, France; biobank registration no. BB‐0033‐00078). All participants gave informed consent before taking part in the study. Exclusion criteria were as follows: neoadjuvant chemotherapy or radiotherapy, macroscopically incomplete resection (R2), tumor histology other than PDAC, and insufficient tumor material available for research. OS was defined as the time interval between the day of surgical resection and death or the date of the last follow‐up, at which point data were censored. Furthermore, one patient who died within 30 days following surgery was excluded from survival analysis. Resected PDAC FFPE tumor samples were used after ethical approval was obtained for the use of patient tumor samples [10].

### Public patient data set comparison

PDX RNAseq (*n* = 29) data for murine‐stromal and human‐epithelial gene expression measures were retrieved from a previous study [24] and are accessible at ArrayExpress repository E‐MTAB‐5039: (https://www.ebi.ac.uk/arrayexpress/experiments/E-MTAB-5039/). The Puleo data set was retrieved from Puleo *et al* [28] and comprised 309 resected patients' microarray profiles. International Cancer Genome Consortium (ICGC) microarray gene expression data sets were downloaded from the ICGC data portal (dcc.icgc.org, release 20). TCGA data were downloaded through the Broad Institute TCGA Genome Data Analysis Center (GDAC) firehose tool (gdac.broadinstitute.org, 20,160,411 data snapshot). The Moffitt data set was downloaded from the entries in the GEO data set GSE71729 [29]. The Grünwald data set was downloaded from the entries in the GEO data set GSE166571 [14].

### Histochemistry and immunohistochemistry

Whole sections of tumors were obtained from resected chemotherapy‐naive PDAC FFPE samples from the Beaujon patient cohort and KPC mice. All the slides of the human cases were reviewed by a pathologist with expertise in pancreatic tumors (JC) to select one representative block. Hematoxylin–eosin–saffron and Picro‐Sirius Red staining was performed on an automated platform (Benchmark Ultra, Ventana, Tucson, AZ, USA). Details of antibodies used are provided in supplementary material, Table S3. Stained slides were scanned using a computer‐controlled capture device (AT turbo, Aperio; Leica Biosystems, Nanterre, France) and quantified using Halo software (Indica Labs, Albuquerque, NM, USA). For Picro‐Sirius Red, the percentage of positive red pixels in the tumor area was computed after visual inspection of the threshold (JC). For αSMA, pan‐cytokeratin (Pan‐CK), CD163, and CD8, the percentage of positive brown pixels in the tumor area was computed after visual inspection of the thresholds (JC). Slides were examined and scored visually by two observers (JC, CN) for the remaining immunostains (POSTN, MYH11, and PDPN). Internal positive controls were smooth muscle in vessels and bowel walls for MYH11 and nerves and lymphatics for PDPN. Moderate or strong staining in >50% of stromal surface defined high POSTN expression, whereas high MYH11 and high PDPN expression were defined as the presence of strong stromal staining, as described previously [10].

### Multiplex immunofluorescence

Tumor sections from whole blocks were obtained from nine resected PDAC FFPE samples from the Beaujon patient cohort and four PDX (*N* = 2 POSTN‐high and *N* = 2 POSTN‐low).  Slides were subjected to antigen retrieval (Tris‐EDTA pH 8.8) and autoclaved at 100 °C for 12 min. Sections were blocked in humidified chamber using bovine serum albumin (3%) for 30 min at RT. Slides were incubated with primary antibodies overnight at 4 °C: anti‐periostin combined with anti‐α‐SMA, or anti‐proliferating cell nuclear antigen (PCNA) alone. Slides were washed in PBS and incubated with secondary antibodies for 1 h at RT. Stained slides were then incubated with an Alexa647‐conjugated pan‐cytokeratin antibody. After PBS washes and incubation with DAPI (15 min, RT), slides were mounted in Fluorescence Mounting Medium (Agilent Dako, Santa Clara, CA, USA) and images acquired using an LSM780 confocal microscope (Zeiss, Zen 2012 blue edition, Jena, Germany) (https://www.zeiss.fr/microscopie/produits/microscope-software/zen-lite.html). Quantification was performed using ImageJ software (https://imagej.nih.gov/ij/download.html) on multiple tissue sections (as indicated in figure legends). See supplementary material, Table S4, for antibody details.

### RNA extraction and sequencing

Total RNA was extracted from FFPE sections using a high‐purity FFPE RNA isolation kit (Roche, Basel, Switzerland) following the manufacturer's protocol. RNA yield and quality were determined using a NanoDrop One spectrophotometer (Thermo Fisher Scientific, Waltham, MA, USA), and fragment size was analyzed using an RNA ScreenTape assay run on a 4200 Bioanalyzer (Agilent Technologies, Santa Clara, CA, USA). DV200 values representing the percentage of RNA fragments above 200 nucleotides in length were estimated, and cases with DV200 more than 30% were included for library preparation.

Library preparation was performed using QuantSeq 3’ mRNA‐Seq REV (Lexogen GmbH, Vienna, Austria) with an input of 150 ng of total FFPE RNA. The pool was sequenced on a NovaSeq 6000 system flow cell SP (Illumina, San Diego, CA, USA) using a 75‐cycle, paired‐end protocol providing approximately 10 million reads per sample. Base call files were converted to fastq format using Bcl2Fastq (Illumina). All RNAseq reads were aligned to the human reference genome (GRCh37, hg19) using STAR (version 2.6.1a_08‐27), quantified using FeatureCount, and Upper‐Quartile normalized.

### RNAseq analysis

A CAF subtype gene set signature was derived for POSTN‐positive (subtype A), MYH11‐positive (subtype B), and PDPN‐positive (subtype C) CAFs using the following approach. Differential gene expression (DGE) analysis was performed on both the CAF cultures, comparing one subtype to all others, and the tumor tissues, comparing CAF marker–positive tumors versus others. DGE was performed using limma following the voom transformation [30]. Gene signatures were defined by the sets of genes that were found to be significantly overexpressed in both *in vitro* CAF cultures and *in situ* tumor tissue (alpha 5% and logfold‐change >0).

The three CAF subtype gene set signatures (POSTN‐, MYH11‐, and PDPN‐RNAsign) were used to quantify each CAF subtype in each of the transcriptome data sets using the average of gene expression z‐score. Basal‐like and classical phenotypes were quantified on tumor profiles or on the epithelial compartment of PDX using the original independent component analysis from the Puleo *et al* [28] study by computing a projection of every sample on the components.

Immune deconvolution tools were applied to PDX stromal compartment and whole tumor transcriptome profiles; XCELL and MCPcounter as available from the immunedeconv package [31]. Gene set enrichment analyses (GSEA) were performed using the fast‐GSEA implementation from Bioconductor (fgsea) using 1,000 permutations [32]. Normalized enrichment scores (NESs) were used for heatmap representation. Single‐gene expression levels (e.g. MKI67) were analyzed using log2 + 1 upper‐quartile normalized counts.

### Single‐cell analysis

To compare our built CAF signatures distinguishing CAF subtypes, we downloaded single‐cell RNAseq raw data from a recent publication [33]. Using the Seurat R package [34], data preprocessing functions were run with default parameters. Low‐quality cells (<200 genes/cell, <3 cells/gene, and >5% mitochondrial genes) and cells from the normal tissue counterpart were excluded. The Seurat package implemented in R was applied to identify major cell types. Highly variable genes were generated and used to perform principal component analysis (PCA). Significant principal components were determined using JackStraw. Cell clusters were projected onto t‐distributed stochastic neighbor embedding (t‐SNE) analysis, using previously computed principal components. Cell‐type identification was based on established marker genes [35] (supplementary material, Table S5). ‘Fibroblast’ cell type was subselected for further analyses. As previously, cell clustering was run and projected onto t‐SNE. CAF signatures were applied to those 6,569 cells using the Gene Set Variation Analysis (GSVA) R package [36] with default parameters. Result representations were done using ggplot2. Comparisons between CAF signatures and gene expression were processed using Pearson's correlation.

### Statistical analyses

Wilcoxon tests were used to compare two groups for continuous variables. Nonparametric one‐way ANOVAs using Kruskal–Wallis tests were performed to compare more than two groups. Survival curves were estimated using the Kaplan–Meier method and compared using the log‐rank test. The level of significance for all tests was *p* < 0.05. Data were analyzed using Prism software version 9.3.1 (GraphPad Software, San Diego, CA, USA).

## Results

### CAF subtype RNAseq signatures correlate with immunostainings and have an assessable prognostic impact

**Figure 1:**
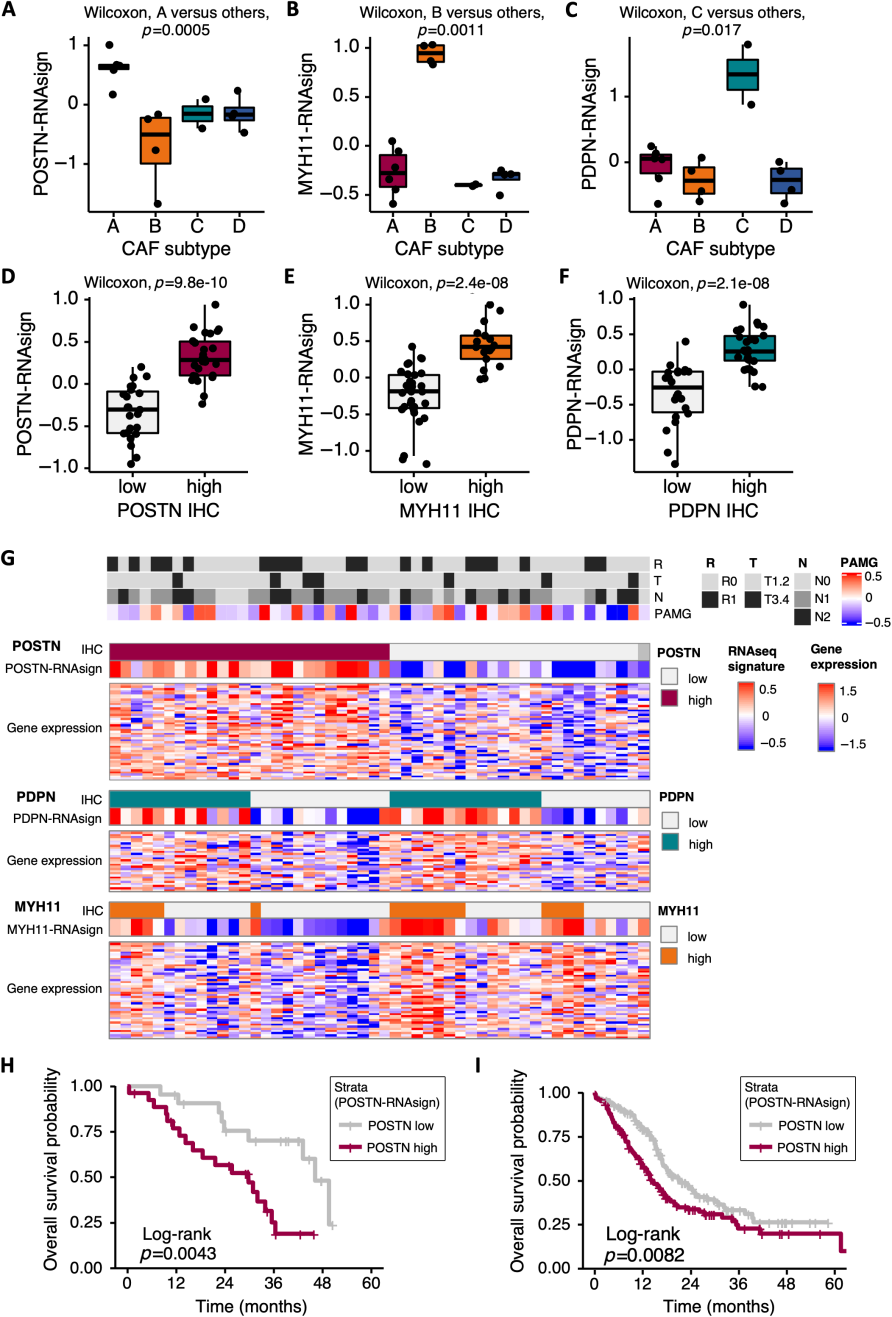
Pancreatic ductal adenocarcinoma (PDAC) cancer‐associated fibroblast (CAF) RNAseq signatures. (A) Periostin (POSTN) multigene RNAseq signature (POSTN‐RNAsign) expression level according to CAF subtypes (A–D, pCAFassigner) in primary CAF cultures (Set 1 [10], *n* = 16). Subtype A is displayed in red, B in orange, C in green, and D in blue. (B) Myosin‐11 (MYH11) multigene RNAseq signature (MYH11‐RNAsign) expression level according to CAF subtypes (A–D, pCAFassigner) in primary CAF cultures (Set 1, *n* = 16). (C) Podoplanin (PDPN) multigene RNAseq signature (PDPN‐RNAsign) expression level according to CAF subtypes (A–D, pCAFassigner) in primary CAF cultures (Set 1, *n* = 16). (D) POSTN‐RNAsign expression level according to POSTN protein expression assessed by immunohistochemistry (IHC) (high in red versus low in gray) in resected human PDAC samples (Beaujon cohort, *n* = 50). (E) MYH11‐RNAsign expression level according to MYH11 protein expression assessed by IHC (high in orange versus low in gray) in resected human PDAC samples (Beaujon cohort, *n* = 50). (F) PDPN‐RNAsign expression level according to PDPN protein expression assessed by IHC (high in green versus low in gray) in resected human PDAC samples (Beaujon cohort, *n* = 50). (G) Heatmap showing POSTN, PDPN, and MYH11 protein expression by IHC (high versus low), multigene RNAseq signatures and gene expression, and association with tumor prognostic features, in individual patients from Beaujon cohort (*n* = 50). Each column represents one patient. POSTN‐high expression by IHC is displayed in red, PDPN‐high expression in green, and MYH11‐high expression in orange, with low expressions in light gray. For gene expression and signatures, higher expression is shown in red and lower expression in blue. Tumor features are displayed in the upper part of the figure: R: resection margin status (R0, no microscopic invasion, in gray and R1, presence of microscopic invasion, in black), T: TNM T‐stage (tumor) (T1‐2 in gray and T3‐4 in black), N: TNM N‐stage (lymph nodes) (N0 in gray, 1 in dark gray, N2 in black); PAMG, pancreatic adenocarcinoma molecular gradient [37] (more classical in blue, more basal‐like in red); NA, not assessable. (H) Kaplan–Meier curves for overall survival (OS) in Beaujon cohort (*n* = 49), according to POSTN‐RNAsign expression (high in red versus low in gray). (I) Kaplan–Meier curves for OS in International Cancer Genome Consortium (ICGC) data set (*n* = 267), according to POSTN‐RNAsign expression (high in red versus low in gray).

To refine our published NanoString‐based CAF subtype transcriptomic signatures (pCAFassigner [10]) and develop signatures that could be transposed to bulk tumor tissue samples, we performed RNAseq analyses. First, we applied RNAseq on a set of 16 primary CAF cultures previously characterized by pCAFassigner [10]. Then we applied RNAseq to a series of 50 resected human PDAC FFPE samples (Beaujon data set) that had been previously classified by IHC into high or low for POSTN, MYH11, and PDPN expression, corresponding to CAF subtypes A, B, and C, respectively [10]. A generalizable multigene expression RNAseq signature for each of the three CAF subtypes, for which an IHC marker was available (i.e. subtypes A, B, and C), was defined by the *in vitro* and *in situ* intersection of CAF‐subtype specific genes and named POSTN‐RNAsign, MYH11‐RNAsign, and PDPN‐RNAsign, respectively (supplementary material, Table S1). We confirmed that each multigene expression RNAseq signature showed a selective enrichment in CAF primary cultures of subtype A (for POSTN‐RNAsign, *p* = 0.0005), subtype B (for MYH11‐RNAsign, *p* = 0.0011), or subtype C (for PDPN‐RNAsign, *p* = 0.017), respectively (Figure 1A–C). Consistently, multigene expression RNAseq signatures correlated well with IHC in patient tumor tissues (scored as previously described [10]), with an enrichment of POSTN‐RNAsign, MYH11‐RNAsign, and PDPN‐RNAsign in IHC‐assigned POSTN‐high (*p* = 9.8e‐10), MYH11‐high (*p* = 2.4e‐08), and PDPN‐high tumors (*p* = 2.1e‐08), respectively (Figure 1D–F). At the individual patient level (*n* = 50), multigene RNAseq signatures were also correlated with protein IHC expression of POSTN, PDPN, or MYH11, as shown in the correlative heatmap (Figure 1G). There was no significant association between RNAseq signatures and classical prognostic factors, i.e. tumor T stage, N stage, or margin R status, or with pancreatic adenocarcinoma molecular gradient (PAMG) defined according to the classical and basal‐like tumor cell transcriptomic subtypes [37] (Figure 1G).

Consistent with the previously described poor prognosis associated with POSTN expression using IHC [10], we showed that tumors rich in POSTN‐positive CAFs based on POSTN‐RNAsign were associated with significantly shorter patient OS, in two human PDAC databases, i.e. our in‐house Beaujon data set (*n* = 49, *p* = 0.0043) (Figure 1H) and in the published ICGC data set (*n* = 267, *p* = 0.0082) (Figure 1I).

Overall, these refined multigene expression RNAseq signatures of CAF subtypes correlated well with IHC, and enrichment in POSTN‐positive CAFs was associated with poor prognosis. Hence, we sought to explore the underlying biological mechanisms of the poor prognosis associated with POSTN‐positive CAFs.

### POSTN‐positive CAFs are characterized by high proliferation and protein synthesis rates and low αSMA expression and are found in peri‐/pretumoral areas

**Figure 2:**
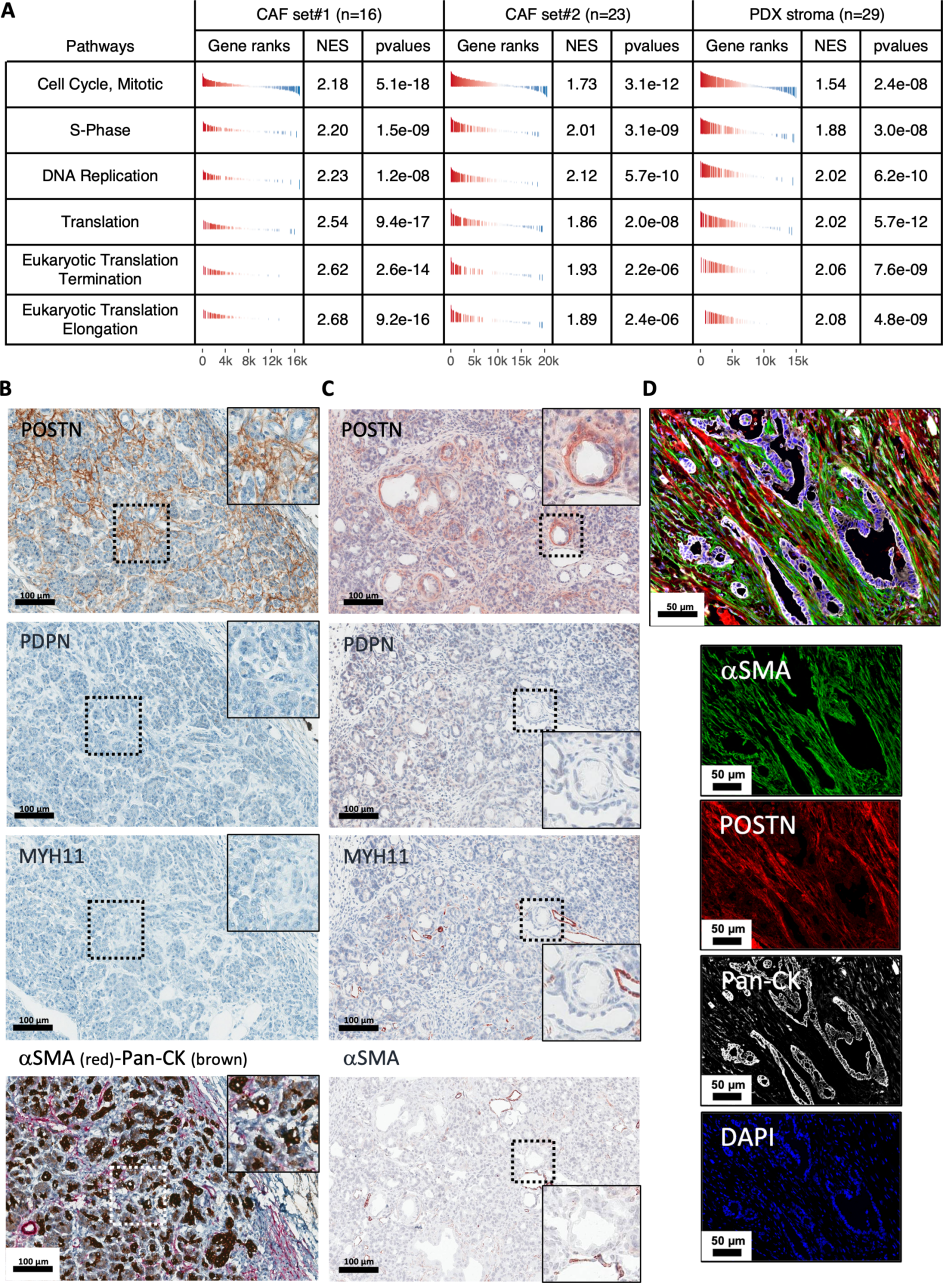
Characterization of periostin‐positive (POSTN‐positive) cancer‐associated fibroblasts (CAFs) *in vitro* and *ex vivo*. (A) Gene set enrichment analysis (GSEA) from RNAseq data of primary CAF cultures (Set 1, *n* = 16 and Set 2, *n* = 23) and patient‐derived xenograft (PDX) stroma (murine genes, *n* = 29) for tumors rich in POSTN‐positive CAFs versus others (according to POSTN‐RNAsign expression). (B) Immunohistochemical (IHC) staining for periostin (POSTN, in brown), podoplanin (PDPN, in brown), myosin‐11 (MYH11, in brown), and costaining for α‐smooth muscle actin (αSMA, in red) and pan‐cytokeratin (Pan‐CK, in brown) on serial sections in adjacent tissue of human tumor from a resected pancreatic ductal adenocarcinoma (PDAC) sample. High magnification (box) highlights a peritumoral pancreatitis area. Low magnification: scale bar: 100 μm. Positive internal control MYH11: muscle cells in artery wall; PDPN nerves. (C) IHC stainings for POSTN, PDPN, MYH11, and αSMA (in brown) on serial sections of preneoplastic area from a KPC mouse. Low magnification: scale bar: 100 μm. High magnification (box) highlights an acinar‐to‐ductal metaplasia (ADM) lesion. (D) Multiplex immunofluorescence (IF) costaining of αSMA (green), POSTN (red), Pan‐CK (white), and DAPI (blue) from resected human PDAC sample. Merged picture (upper panel) and individual stainings (lower panels). Scale bar: 50 μm.

First, we used GSEA analysis of RNAseq data from two independent sets of primary CAF cultures (Set 1, *n* = 16, and Set 2, *n* = 23) to describe the pathways enriched in POSTN‐positive CAFs (Figure 2A). In both sets, we found an enrichment in pathways associated with cell proliferation and protein synthesis (i.e. mRNA translation) in this CAF subtype, consistent with our previous findings that subtype A CAFs from pCAFassigner are more proliferative than other CAF subtypes [10] and that expression of POSTN correlates with high protein synthesis rates in human and murine CAFs [38]. The association between POSTN‐RNAsign and high proliferation rate was also validated in primary CAF cultures using Ki67 (*MKI67*) RNA expression (from RNAseq, Set 1) (supplementary material, Figure S1A) and cell counting (Set 2) (supplementary material, Figure S1B). In addition, we confirmed these observations *in vivo* by performing GSEA analysis of RNAseq data from the murine (i.e. stromal, including CAFs) compartment of PDX models consisting in the subcutaneous engraftment of a large panel (*n* = 29) of patient tumors [24], showing significant enrichment in these pathways (Figure 2A). Then, using IHC, we identified a specific pattern of POSTN expression in peri‐/pretumoral areas. In human PDAC samples, POSTN‐expressing CAFs were observed in pancreatitis areas adjacent to tumors (Figure 2B and supplementary material, Figure S1C). Moreover, in the KPC mouse model, POSTN expression was localized around preneoplastic acinar‐to‐ductal metaplasia (ADM) and pancreatic intraepithelial neoplasia (PanIN) lesions (Figure 2C and supplementary material, Figure S1D). Conversely, αSMA, MYH11, and PDPN expression was low or null in the fibroblasts from these areas (Figure 2B,C and supplementary material, Figure S1C,D). For αSMA in KPC mice, it was not expressed surrounding ADM and inconsistently expressed surrounding PanIN lesions. As previously described using western blotting [10], multiplex co‐IF staining revealed that most POSTN‐high CAFs were αSMA‐low (94.5%, whole tumor area analysis from *N* = 9 patients) (Figure 2D and supplementary material, Figure S1E,F).

Taken together with our previous finding that PSCs cultured with conditioned media from cancer cells induces a transition from POSTN‐high to POSTN‐low subtypes [10], these data suggested that POSTN‐positive/αSMA‐low CAFs may be the initial reactive CAFs emerging at early steps of PDAC carcinogenesis from the peri‐acinar PSCs.

### POSTN‐positive CAFs are associated with highly cellular tumors and with macrophage infiltrates

**Figure 3:**
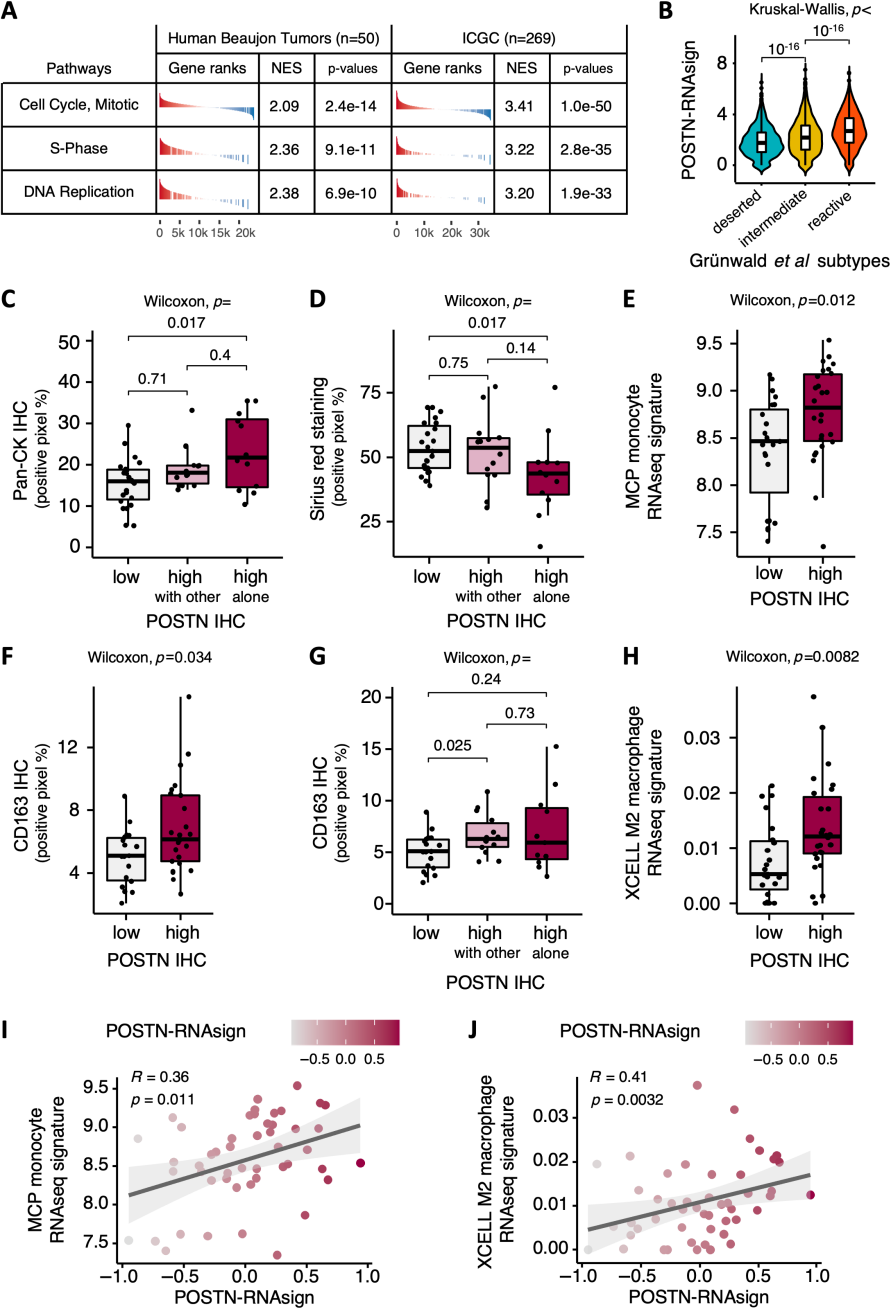
Characterization of tumors rich in periostin‐positive (POSTN‐positive) cancer‐associated fibroblasts (CAFs). (A) Gene set enrichment analysis (GSEA) from RNAseq data of bulk human tumors from Beaujon cohort (*n* = 50) and International Cancer Genome Consortium (ICGC) data set (*n* = 269), for tumors rich in POSTN‐positive CAFs versus others (according to POSTN‐RNAsign expression). (B) Expression of POSTN‐RNAsign according to ‘reactive’, ‘intermediate’, or ‘deserted’ subtumor microenvironment areas from Grünwald *et al* [14] data set. (C) Quantification of immunohistochemical (IHC) staining for pan‐cytokeratin (Pan‐CK, percentage of positive pixels) according to POSTN protein expression (POSTN‐low in gray, POSTN‐high with MYH11, or PDPN coexpression in pink, and POSTN‐high without MYH11/PDPN coexpression in red) assessed by IHC in resected human pancreatic ductal adenocarcinoma (PDAC) samples (Beaujon cohort, *n* = 50). (D) Quantification of Picro‐Sirius Red (‘Sirius red’) stained area (percentage of positive pixels) according to POSTN protein expression (POSTN‐low in gray, POSTN‐high with MYH11, or PDPN coexpression in pink, and POSTN‐high without MYH11/PDPN coexpression in red) assessed by IHC in resected human PDAC samples (Beaujon cohort, *n* = 50). (E) Quantification of microenvironment cell populations (MCP) monocyte RNAseq signature expression level according to POSTN protein expression (high in red versus low in gray) assessed by IHC in resected human PDAC samples (Beaujon cohort, *n* = 50). (F) Quantification of IHC staining for CD163 (percentage of positive pixels) according to POSTN protein expression (high in red versus low in gray) assessed by IHC in resected human PDAC samples (Beaujon cohort, *n* = 50). (G) Quantification of IHC staining for CD163 (percentage of positive pixels) according to POSTN protein expression (POSTN‐low in gray, POSTN‐high with MYH11 and/or PDPN coexpression in pink, and POSTN‐high without MYH11/PDPN coexpression in red) assessed by IHC in resected human PDAC samples (Beaujon cohort, *n* = 50). (H) Quantification of XCELL M2 macrophage RNAseq signature expression level according to POSTN protein expression (high in red versus low in gray) assessed by IHC in resected human PDAC samples (Beaujon cohort, *n* = 50). (I) Correlation between POSTN multigene RNAseq signature (POSTN‐RNAsign) expression and MCP monocyte RNAseq signature expression in resected human PDAC samples (Beaujon cohort, *n* = 50). (J) Correlation between POSTN multigene RNAseq signature (POSTN‐RNAsign) expression and XCELL M2 macrophage RNAseq signature expression in resected human PDAC samples (Beaujon cohort, *n* = 50).

We next characterized the phenotype of tumors rich in POSTN‐positive CAFs. First, using GSEA of two independent bulk RNAseq data sets of resected human PDAC samples (in‐house/Beaujon, *n* = 50, and public/ICGC, *n* = 269), we showed that proliferation pathways were enriched in tumors with high abundance of POSTN‐positive CAFs, as defined using POSTN‐RNAsign (Figure 3A). This could reflect increased proliferation in the stromal or tumoral compartment. To address this point, we performed co‐immunostaining for PCNA (marker of cell proliferation [39]), pan‐cytokeratin (Pan‐CK, highlighting tumor cells), and DAPI (for nuclei) in *N* = 2 POSTN‐high and *N* = 2 POSTN‐low PDX tumors and quantified PCNA expression in stromal and tumoral cells. It showed that cell proliferation was higher in the stromal compartment of the POSTN‐high tumors versus POSTN‐low tumors (33.8 versus 18.2%), while no difference was observed in tumor cells (71.9 versus 69.5%). This result confirmed that PDX is a reliable model to recapitulate the stromal phenotype from PDAC human tumors [24]. In addition, we showed that POSTN‐RNAsign was enriched in ‘reactive’ sub‐tumor microenvironment (TME) areas as opposed to ‘deserted’ ones from the Grünwald *et al* [14] data set (Figure 3B). Taken together, these findings suggest that the enrichment in proliferation signatures in bulk samples (i.e. pooling together stromal and tumor epithelial signatures, in the Beaujon or ICGC data set) from tumors rich in POSTN‐positive CAFs is attributable to high cellular proliferation rate in the stromal (i.e. CAF or immune cells) (Figure 2A) rather than the tumor epithelial compartment.

POSTN‐high tumors (as assessed by IHC) were associated with higher tumor cell‐to‐stroma ratio, as evidenced by high relative Pan‐CK immunostaining (*p* = 0.011) (supplementary material, Figure S2A), and low abundance of collagen, specifically for ‘pure’ POSTN‐high tumors (POSTN high alone, i.e. expressing POSTN but not PDPN or MYH11 by IHC) (POSTN‐high alone versus POSTN‐low, Pan‐CK, *p* = 0.017, Figure 3C, and Picro‐Sirius Red staining, *p* = 0.017, Figure 3D). There was a nonsignificant trend for an enrichment in basal‐like tumor subtype (RNA signature) in POSTN‐high tumors (POSTN expression assessed by IHC, *p* = 0.095 or POSTN‐RNAsign*, p* = 0.056) (supplementary material, Figure S2B,C).

Interestingly, POSTN‐high tumors were associated with an enrichment in monocyte RNA signature (*p* = 0.012) (Figure 3E). The presence of macrophages was confirmed by CD163 immunostaining (M2 macrophage marker, *p* = 0.034, Figure 3F), with an enrichment particularly when POSTN expression was combined with PDPN or MYH11 expression (POSTN‐high with other versus POSTN‐low, *p* = 0.025, Figure 3G), and by XCELL M2 macrophage RNA signature (*p* = 0.0082) [40] (Figure 3H). Moreover, we observed a positive correlation between POSTN‐RNAsign and monocyte and M2 macrophage RNA signatures (*R* = 0.36, *p* = 0.011 and *R* = 0.41, *p* = 0.0032, respectively) (Figure 3I,J). In contrast, there was no association with T cell RNA signature and CD8 immunostaining (supplementary material, Figure S2D–F) or with myeloid dendritic cell (mDC) RNA signatures (supplementary material, Figure S2G,H).

Overall, POSTN‐positive CAF was associated with tumors with high epithelial cellularity and low stroma abundance and with infiltration by inflammatory M2 macrophages.

### PDPN‐positive CAFs are associated with immune‐related signatures and recruitment of dendritic cells

Previously [10], we demonstrated, using NanoString technology, that CAF subtype C from pCAFassigner, which expressed a high level of PDPN, was associated with unique immune‐related pathways.

**Figure 4:**
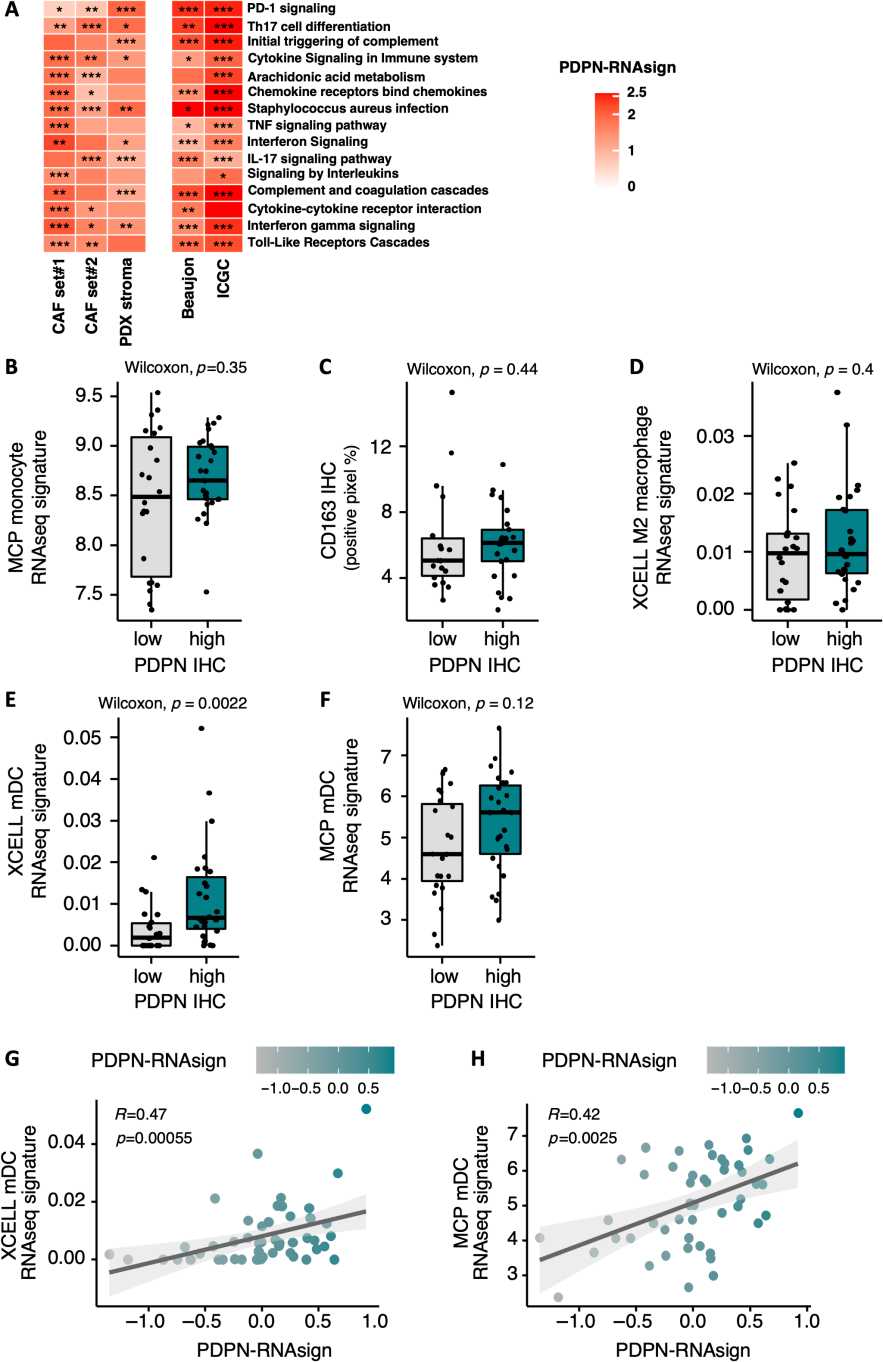
Characterization of tumors rich in podoplanin‐positive (PDPN‐positive) cancer‐associated fibroblasts (CAFs). (A) Gene set enrichment analysis (GSEA) from RNAseq data of primary CAF cultures (Set 1, *n* = 16, and Set 2, *n* = 23), patient‐derived xenograft (PDX) stroma (mouse genes, *n* = 29), and bulk human tumor samples from Beaujon cohort (*n* = 50), and International Cancer Genome Consortium (ICGC) data set (*n* = 267), for tumors rich in PDPN‐positive CAFs versus others. (B) Quantification of microenvironment cell populations (MCP) monocyte RNAseq signature expression level according to podoplanin (PDPN) protein expression (high in green versus low in gray) assessed by IHC in resected human pancreatic ductal adenocarcinoma (PDAC) samples (Beaujon cohort, *n* = 50). (C) Quantification of IHC staining for CD163 (percentage of positive pixels) according to PDPN protein expression (high in green versus low in gray) assessed by IHC in resected human PDAC samples (Beaujon cohort, *n* = 50). (D) Quantification of XCELL M2 macrophage RNAseq signature expression level according to PDPN protein expression (high in green versus low in gray) assessed by IHC in resected human PDAC samples (Beaujon cohort, *n* = 50). (E) Quantification of XCELL mDC (dendritic cells) RNAseq signature expression level according to PDPN protein expression (high in green versus low in gray) assessed by IHC in resected human PDAC samples (Beaujon cohort, *n* = 50). (F) Quantification of MCP mDC RNAseq signature expression level according to PDPN protein expression (high in green versus low in gray) assessed by IHC in resected human PDAC samples (Beaujon cohort, *n* = 50). (G) Correlation between PDPN multigene RNAseq signature (PDPN‐RNAsign) expression and XCELL mDC RNAseq signature expression in resected human PDAC samples (Beaujon cohort, *n* = 50). (H) Correlation between PDPN multigene RNAseq signature (PDPN‐RNAsign) expression and MCP mDC RNAseq signature expression in resected human PDAC samples (Beaujon cohort, *n* = 50).

GSEA of RNAseq data from the two independent sets of CAF primary cultures (Set 1, *n* = 16, and Set 2, *n* = 23), from PDX stroma (*n* = 29), and from bulk resected human PDAC samples (Beaujon, *n* = 50, and ICGC, *n* = 269), revealed that tumors rich in PDPN‐positive CAFs, as defined with PDPN‐RNAsign, are also significantly enriched in multiple immune‐related pathways, involved in both innate (e.g. Toll‐like receptors) and adaptive (e.g. PD‐1, interferon gamma) immunity (Figure 4A).

Regarding immune cells, although PDPN‐positive CAFs displayed immune‐related signatures, PDPN‐high tumors (as assessed by IHC) were not significantly associated with monocyte/macrophage RNA signatures or CD163 immunostaining (Figure 4B–D) or with T cell RNA signature or CD8 immunostaining (supplementary material, Figure S3A,B). In contrast, tumors rich in PDPN‐positive CAFs were associated with an enrichment in mDC RNA signatures, when using either PDPN IHC (*p* = 0.0018 for XCELL signature) (Figure 4E,F) or the PDPN‐RNAsign, with a positive correlation (*R* = 0.47, *p* = 0.00055 and *R* = 0.42, *p* = 0.0025 for XCELL and microenvironment cell populations (MCP) mDC signatures, respectively) (Figure 4G,H).

Considered on its own, PDPN expression by IHC was not associated with ECM abundance (supplementary material, Figure S3C). There was a nonsignificant trend for an enrichment in classical tumor subtype in PDPN‐high tumors by IHC (*p* = 0.076) and a significant yet low correlation using PDPN‐RNAsign (*R* = 0.29, *p* = 0.046) (supplementary material, Figure S3D,E).

Overall, PDPN‐positive CAFs were associated with an enrichment in immune‐related signatures and recruitment of mDC, but without impact on macrophage or T cell infiltrates.

### POSTN‐positive and PDPN‐positive CAF combinations define specific tumor microenvironments

**Figure 5:**
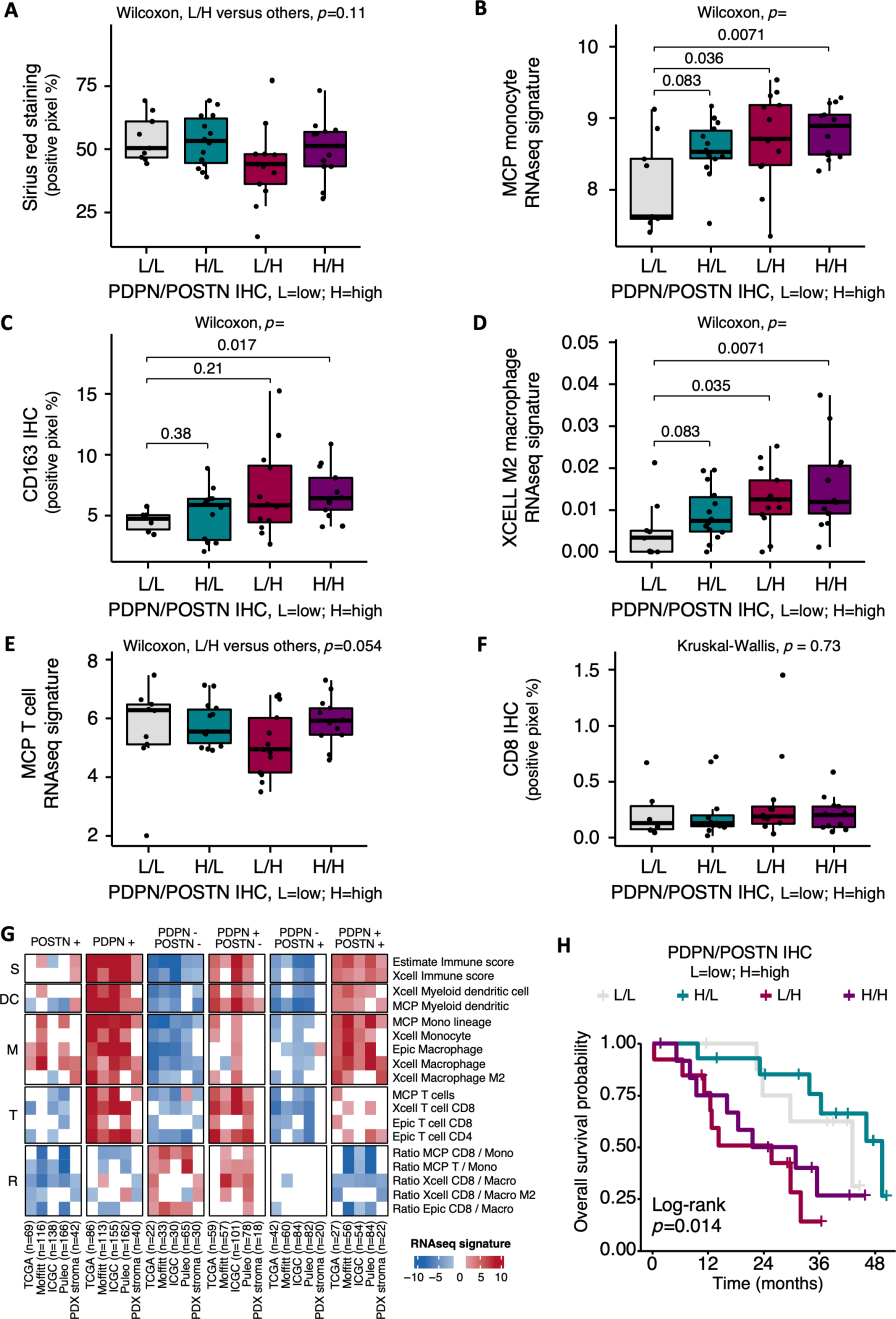
Combination of periostin (POSTN)‐ and podoplanin (PDPN)‐positive cancer‐associated fibroblasts (CAFs). (A) Quantification of Picro‐Sirius Red (‘Sirius red’) stained area (percentage of positive pixels) according to POSTN and PDPN combined protein expressions assessed by immunohistochemistry (IHC) in resected human PDAC samples (Beaujon cohort, *n* = 50). Low PDPN/low POSTN tumors are displayed in gray, high PDPN/low POSTN in green, low PDPN/high POSTN in red, high PDPN/high POST in purple. (B) Quantification of microenvironment cell populations (MCP) monocyte RNAseq signature expression level according to POSTN and PDPN combined protein expressions assessed by IHC in resected human PDAC samples (Beaujon cohort, *n* = 50). (C) Quantification of IHC staining for CD163 (percentage of positive pixels) according to POSTN and PDPN combined protein expressions assessed by IHC in resected human PDAC samples (Beaujon cohort, *n* = 50). (D) Quantification of XCELL M2 macrophage RNAseq signature expression level according to POSTN and PDPN combined protein expressions assessed by IHC in resected human PDAC samples (Beaujon cohort, *n* = 50). (E) Quantification of MCP T cell RNAseq signature expression level according to POSTN and PDPN combined protein expressions assessed by IHC in resected human PDAC samples (Beaujon cohort, *n* = 50). (F) Quantification of IHC staining for CD8 (percentage of positive pixels) according to POSTN and PDPN combined protein expressions assessed by IHC in resected human PDAC samples (Beaujon cohort, *n* = 50). (G) Heatmap summarizing quantification of immune cell RNAseq signatures according to POSTN and PDPN multigene RNAseq signatures in five different data sets (TCGA, Moffitt, ICGC, Puleo, PDX). Higher expression is shown in red and lower expression in blue. (H) Kaplan–Meier curves for overall survival in Beaujon cohort (*n* = 49), according to combined POSTN and PDPN expression by IHC.

We assessed tumor features when combining POSTN‐positive and PDPN‐positive CAF status (i.e. by classifying the tumors into four groups). We observed that tumors with PDPN‐low/POSTN‐high expression (as assessed by IHC) tended to display the lowest ECM abundance (Picro‐Sirius Red, *p* = 0.11) (Figure 5A). In addition, tumors showing PDPN‐high/POSTN‐high expression were the most consistently infiltrated by macrophages (as assessed with the monocyte/macrophage RNA signatures, *p* = 0.0071, or using the CD163 IHC marker, *p* = 0.017), suggesting a combined effect of the two CAF subpopulations (Figure 5B–D). In addition, there was a trend for less T cell infiltration in PDPN‐low/POSTN‐high tumors (*p* = 0.054) (Figure 5E), which was not related to CD8 T cell infiltrates assessed by IHC (Figure 5F).

We further explored, in five RNAseq databases, representing a total of 934 PDAC patient tumors (TCGA, Moffitt *et al* [29], ICGC, Puleo *et al* [28]) and 29 PDXs [24], the differential enrichment of several immune cell type RNA signatures according to the combination of POSTN‐positive CAFs and PDPN‐positive CAFs, as defined using the POSTN‐RNAsign and PDPN‐RNAsign (Figure 5G and supplementary material, Table S2). Global immune score (i.e. composite score of immune cell types) was the highest in tumors rich in PDPN‐positive CAFs (PDPN+), this subtype being a prerequisite for the presence of immune cells (absent in PDPN− tumors, whatever the status of POSTN). Interestingly, in tumors rich in PDPN‐positive CAFs (PDPN+), the coenrichment in POSTN‐positive CAFs is required to reach the highest levels for the infiltration with monocytes/macrophages (PDPN+/POSTN+ versus PDPN+/POSTN−). Conversely, POSTN‐positive CAFs alone (PDPN−/POSTN+) were not associated with increased in monocyte/macrophage infiltrates. These findings validated our results obtained in Figure 5B–D and strongly suggested a cooperation between POSTN‐positive and PDPN‐positive CAF populations to induce the recruitment of monocytes/macrophages. Figure 5G further showed that tumors enriched in PDPN‐positive CAFs but with low abundance of POSTN‐positive CAFs (PDPN+/POSTN−) were the most infiltrated with T lymphocytes and mDCs, suggesting that POSTN‐positive CAFs opposed (directly, or indirectly *via* monocytes/macrophages) the recruitment of T cells and mDCs driven by PDPN‐positive CAFs. Moreover, POSTN‐low tumors (PDPN−/POSTN− and PDPN+/POSTN−) both displayed a high CD8:macrophage ratio, even if they differed for immune cell density (low global immune score in PDPN−/POSTN− versus high in PDPN+/POSTN−).

Finally, patient prognosis was driven mainly by POSTN‐positive CAFs (shortest OS in POSTN‐high groups; Beaujon, *n* = 49, by IHC), even if the combination with PDPN‐positive CAFs allowed some refinement in the OS estimation (longest median OS in PDPN‐high/POSTN‐low group and shortest in PDPN‐low/POSTN‐high group; ICGC, *n* = 267, by RNAseq) (Figure 5H and supplementary material, Figure S4).

Overall, these results highlighted that the combination of POSTN‐positive CAFs and PDPN‐positive CAFs defined four groups with specific immune microenvironment and prognosis.

### Our CAF subtypes do not fully overlap with myCAF/iCAF classification

Lastly, we explored the association between our CAF subtypes and previously described classifications.

**Figure 6:**
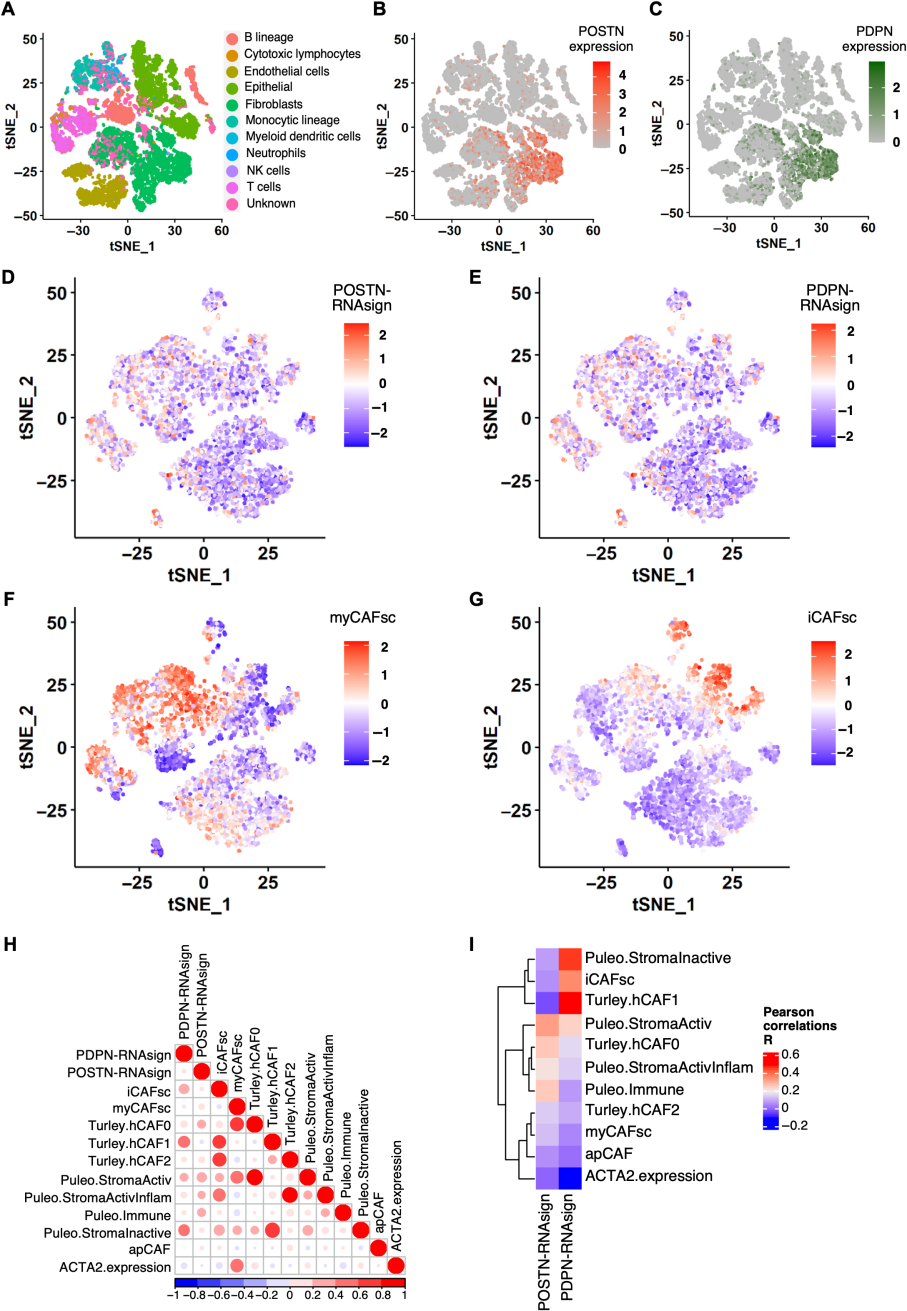
Single‐cell RNA sequencing cross‐analyses with published cancer‐associated fibroblast (CAF) classifications. (A) Plot of different cell types onto t‐distributed stochastic neighbor embedding (t‐SNE) map from single‐cell RNA sequencing. Fibroblasts are highlighted in green. (B) Plot of expression levels of periostin (POSTN) in each analyzed cell (all cell types) onto t‐SNE map. Color key from gray to red indicates relative expression levels from low to high. (C) Plot of expression levels of podoplanin (PDPN) in each analyzed cell (all cell types) onto t‐SNE map. Color key from gray to green indicates relative expression levels from low to high. (D) Plot of POSTN multigene RNAseq signature (POSTN‐RNAsign) expression level in each analyzed cell (fibroblasts only). Higher expression is shown in red and lower expression in blue. (E) Plot of PDPN multigene RNAseq signature (PDPN‐RNAsign) expression level in each analyzed cell (fibroblasts only). Higher expression is shown in red and lower expression in blue. (F) Plot of myofibroblastic CAF (myCAF) (Tuveson's group [12]) RNAseq signature expression level in each analyzed cell (fibroblasts only). Higher expression is shown in red and lower expression in blue. (G) Plot of inflammatory CAF (iCAF) (Tuveson's group [12]) RNAseq signature expression level in each analyzed cell (fibroblasts only). Higher expression is shown in red and lower expression in blue. (H) Pearson's *R* correlation between each published transcriptomic signatures score assessed by GSVA or *ACTA2* expression in the fibroblast subgroup. Positive correlation is shown in red and negative correlation in blue. (I) Pearson's *R* correlation between POSTN‐RNAsign or PDPN‐RNAsign and published transcriptomic signatures and *ACTA2* expression. Positive correlation is shown in red and negative correlation in blue.

Using single‐cell analysis, we showed that POSTN‐RNAsign, PDPN‐RNAsign, and MYH11‐RNAsign highlighted different fibroblast subsets (Figure 6A–C and supplementary material, Figure S5A).

When compared to other CAF classifications (Tuveson's group [12], Turley's group [15], Puleo *et al* [28]), PDPN‐positive CAFs were positively correlated with iCAF, Turley hCAF1 (related to iCAF), and Puleo Inactive Stroma; in contrast, POSTN‐positive CAFs displayed no strong correlation with any described RNA signatures (particularly, myCAF/iCAF signatures) yet showed low positive association with Puleo Activated Stroma, Puleo Immune, and Turley hCAF0 (LRRC15^+^), suggesting that it is an activated fibroblast even if αSMA‐low (Figure 6D–I and supplementary material, Figure S5B–F).

Overall, these results revealed that our CAF signatures identified specific CAF subsets not fully overlapping molecularly with previously described CAF subtypes. PDPN‐positive CAFs identified an iCAF‐like subset whereas POSTN‐positive CAFs were not correlated with the classical myCAF/iCAF classification.

## Discussion

Our results add to the growing evidence that CAFs impact PDAC prognosis and tumor phenotypic heterogeneity. Thanks to the refinement of the gene signatures of our previously described subtype A–C CAFs, we were able to interrogate our in‐house data set (*n* = 50, with extensive IHC characterization) and public bulk RNAseq databases comprising 934 PDAC patients and 29 PDX, to confirm that POSTN‐positive CAFs and PDPN‐positive CAFs were associated with patient OS (in particular, POSTN‐positive CAFs) and TME features (ECM abundance, immune cell infiltrates). Further, we showed that PDPN‐positive CAF resembled the published iCAF subset molecularly (based on immunogenic RNA signatures and single‐cell analysis showing positive correlations between PDPN‐RNAsign and iCAF RNA signature), yet that POSTN‐positive CAF identified a novel type of fibroblast emerging at early steps of PDAC carcinogenesis (detected in adjacent tissue of human tumors and surrounding preneoplastic lesions in KPC), with low αSMA expression, which does not fit with the ‘mature’ myCAF/iCAF phenotypes. Importantly, we showed that the combination of the gene signatures associated with POSTN‐positive CAFs and PDPN‐positive CAFs dictated the presence of specific immune infiltrates, whereby (1) tumors poor in PDPN‐positive CAFs are immune‐cold and (2) tumors rich in PDPN‐positive CAFs include putative antitumor immune infiltrates (presence of T and mDCs ce), except if also rich in POSTN‐positive CAFs, whose associated presence favors macrophage recruitment but excludes T cells. Conversely, the presence of only POSTN‐positive CAFs is not sufficient to recruit macrophages, demonstrating the cooperativity between both subtypes to shape the specific proinflammatory and immunosuppressive PDAC TME.

POSTN is a 90‐kDa secreted ECM protein belonging to the fasciclin family. It functions as an adhesion molecule and mediates its effects through binding to the integrin receptors αvβ3, αvβ5, and α6β4 [41]. The expression level of POSTN in healthy adult tissues is very low, whereas it is found highly upregulated in a wide spectrum of inflammatory diseases and cancers. In pancreatic diseases, POSTN is involved in pancreatic regeneration after acute pancreatitis [42]. Using IHC, we showed that, in addition to being expressed at the invading front of tumors [10], POSTN expression was also found in peritumoral pancreatitis area in human tumors and surrounding preneoplastic lesions (ADMs and PanINs) in genetically engineered KPC mouse models. In addition, we previously showed that PSC ‘cancer education’ induced a transition from POSTN‐high to POSTN‐low subtypes [10]. Taken together, these data suggested that POSTN‐positive CAFs may be the initial reactive CAFs emerging at early steps of PDAC carcinogenesis from the peri‐acinar PSCs. POSTN expression is associated with aggressive tumor features, i.e. with poor prognosis and metastatic dissemination, in a variety of cancers, including pancreatic [43], biliary tract [44], colorectal [45], oesophageal [46], head and neck [47], lung [48], breast [49], ovarian [50], and genito‐urinary [51] carcinomas, and melanoma [52]. Current evidence indicates that CAF‐derived POSTN can remodel TME to form supportive metastatic, perivascular, and cancer stem cell niches by crosstalk with other signaling molecules or by recruiting inflammatory and immune cells [53, 54]. Using our in‐house series of resected human PDAC samples and public ICGC data set, we found that POSTN‐high tumors, assessed by IHC or using RNAseq signature, were associated with (1) shorter OS ([10]), (2) poor prognostic (basal‐like/poorly differentiated) PDAC epithelial subtypes [10], (3) higher tumor cellularity and lower abundance of collagen deposits, particularly when tumors display only POSTN expression, without MYH11 or PDPN expression, and (4) with infiltration by M2 macrophages. Overall, high POSTN expression was associated with poor prognostic features in patients. However, works from other teams using POSTN knockout mice showed that POSTN was required for tumor capsule formation [55]. The growth of tumors that had been grafted into POSTN −/− mice was significantly accelerated compared with that of the same tumors grafted into POSTN +/+ mice. This suggests that some POSTN+ CAFs may be protective against tumor growth. A model conciliating these data with ours would be that aggressive tumor cells trigger rapid and localized PSC activation into POSTN‐positive CAFs, leading to POSTN expression upregulation, probably through TGFβ ligand secretion, and explaining why POSTN‐positive CAFs correlate with poor prognosis. Once these POSTN‐positive CAFs get ‘older’, they may lose POSTN expression, as suggested by our *in vitro* cancer‐educated experiment [10]. Hence, POSTN‐low CAFs might either emerge from POSTN‐positive CAFs in a dynamic process or have a different cellular origin.

We previously published findings that PDPN‐positive CAFs (subtype C CAF from pCAFassigner) were associated with immune‐related signatures [10], which we confirmed here using our refined RNAseq signature (PDPN‐RNAsign) and in enlarged patient cohorts. Inferring our PDPN‐RNAsign to PDAC transcriptomic single‐cell signatures showed that PDPN‐positive CAFs resembled iCAF [12] and hCAF1 [5], and the PDPN‐RNAsign correlated with a global increase in intratumoral infiltration by immune cells. Consistently, a positive association between PDPN‐positive CAFs and lymphocyte infiltration was reported in other cancer types [56], suggesting that PDPN expression in CAFs may be an indicator of immunogenic tumors. When analyzed considering both POSTN‐positive and PDPN‐positive CAFs, we observed that in tumors rich in PDPN‐positive CAFs, POSTN‐positive CAFs, when also present, controlled which specific immune cells were present, restraining T cells but favoring monocytes and immunosuppressive M2 macrophages. Moreover, the ratio of CD8:macrophage appeared to be more important than immune cell density in predicting a favorable prognosis in POSTN‐low tumors, which was consistent with other reports [57, 58]. These results suggest that the PDAC immune phenotype is dictated by the presence of at least two CAF subtypes, which cooperate to control the recruitment or polarization into tumors of lymphocytic and myeloid lineages, the latter being associated with poor prognosis [59].

These findings warrant independent, prospective validation of the proposed inter‐ and intratumoral heterogeneity, dynamics, and prognostic impact in independent cohorts, as well as functional ascertainment *in vitro ex vivo* and murine models of PDAC.

Our results provide evidence for potential CAF‐based patient prognostic stratification. Moreover, CAF subtypes, by their association with basal‐like/classical subtypes, may modulate tumor sensitivity to chemotherapy [60]. CAF subtypes may also be explored as predictive markers of response to immune therapy [61]. In particular, our PDPN‐ and POSTN‐RNAsign signatures may help to stratify patients into responsive (PDPN‐high/POSTN‐low) versus unresponsive (POSTN‐high) patients to immunotherapies. Ultimately, targeting selectively or reprogramming ‘bad’ populations of CAFs (e.g. POSTN+) may emerge as a new therapeutic strategy in PDAC.

## Author contributions statement

Original ideas were from CN and CB. CN, RN, AT‐R, HK, JC and CB designed the work. Data were acquired by CN, RN, JR, AT‐R, AB, LA‐X, SV, FA, MF, MH, RS, CK, AP, VR, MM, IB, JC and CB. Data were analyzed and interpreted by CN, RN, JR, AT‐R, HK, JC and CB. Material was provided by AP, VR, MM, JC, HK and our acknowledged collaborators. The study was supervised by CN and CB. The manuscript was written by CN, RN, JR, AT‐R, LA‐X, JC and CB, and revised and approved by all authors.

## Supporting information


**Figure S1.** Periostin‐positive cancer‐associated fibroblasts (CAFs) are proliferating and are low α‐smooth muscle actin–expressing CAFs localized in peritumoral lesions
**Figure S2.** POSTN‐high tumors present a higher proliferative stroma and are not associated with T‐cell or myeloid dendritic cell RNAseq signatures
**Figure S3.** Podoplanin‐high tumors are associated with the classical subtype RNAseq signature but not with T‐cell RNAseq signatures
**Figure S4.** Kaplan–Meier curves for overall survival (OS) in International Cancer Genome Consortium cohort (*n* = 247), according to combined periostin and podoplanin multigene RNAseq signatures
**Figure S5.** Cancer‐associated fibroblast single‐cell analyses

**Table S1.** Transcripts included in each periostin‐, myosin‐11‐, or podoplanin‐RNAseq signature

**Table S2.** Distribution of samples from the five data sets according to periostin and podoplanin expression (from RNAseq signatures)
**Table S3.** Antibodies used for immunohistochemistry on resected pancreatic ductal adenocarcinoma formalin‐fixed and paraffin‐embedded samples from Beaujon patient cohort and from KPC models
**Table S4.** Antibodies used for multiplex immunofluorescence
**Table S5.** Transcripts used to define cell type clusters for single‐cell analyses

## Data Availability

RNA‐sequencing data were deposited in the open data repository Zenodo under doi 10.5281/zenodo.7064376 and are available for download at the following link: https://doi.org/10.5281/zenodo.7064376.
