# Supplementary material for: Periostin‐ and podoplanin‐positive cancer‐associated fibroblast subtypes cooperate to shape the inflamed tumor microenvironment in aggressive pancreatic adenocarcinoma
Source: J Pathol. 2022 Oct 21;258(4):408–25. doi: 10.1002/path.6011 (PMC9828775; doi:10.1002/path.6011)
Supplement: Supplementary file 1 — Figure S1. Periostin‐positive cancer‐associated fibroblasts (CAFs) are proliferating and are low α‐smooth muscle actin–expressing CAFs localized in peritumoral lesions Figure S2. POSTN‐high tumors present a higher proliferative stroma and are not associated with T‐cell or myeloid dendritic cell RNAseq signatures Figure S3. Podoplanin‐high tumors are associated with the classical subtype RNAseq signature but not with T‐cell RNAseq signatures Figure S4. Kaplan–Meier curves for overall survival (OS) in International Cancer Genome Consortium cohort (n = 247), according to combined periostin and podoplanin multigene RNAseq signatures Figure S5. Cancer‐associated fibroblast single‐cell analyses [file PATH-258-408-s002.docx]

**Periostin- and podoplanin- positive cancer-associated fibroblast subtypes cooperate to shape the inflamed tumor microenvironment in aggressive pancreatic adenocarcinoma**

C Neuzillet *et al. J Pathol* DOI: <https://doi.org/10.1002/path.6011>

**Supplementary Figures S1–S5**

Reference numbers refer to the main text list

**
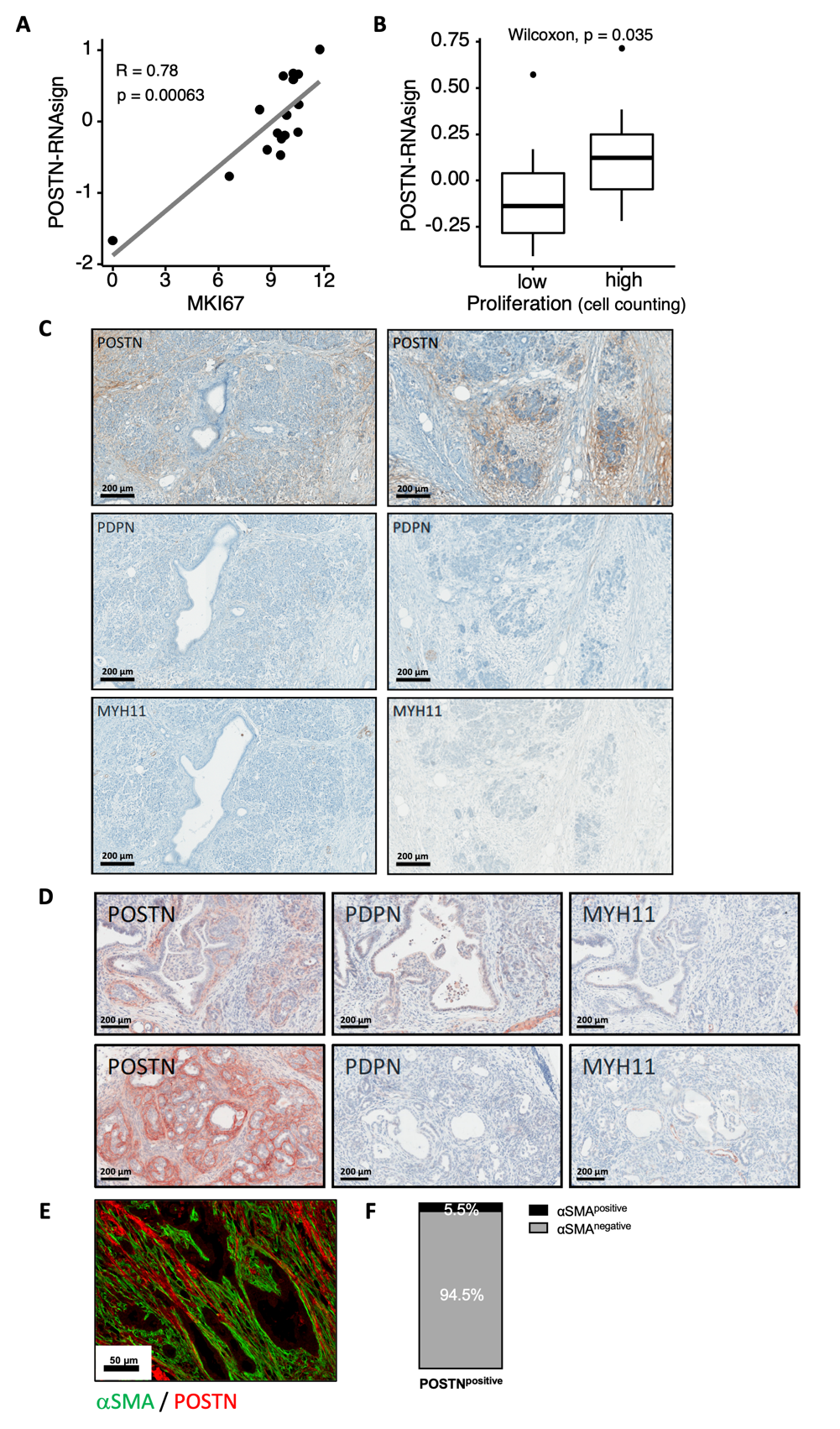
**

**Figure S1. POSTN-positive CAFs are proliferating and are low αSMA-expressing CAFs localized in peri-tumoral lesions**

1. Correlation between periostin (POSTN) multigene signature (POSTN-RNAsign) expression and *MIK67* gene expression by RNAseq in cancer-associated fibroblast (CAF) primary cultures (set #1, n=16).
2. Cell proliferation (assessed by cell counting) according to POSTN-RNAsign expression in CAF primary cultures (set #2, n=23).
3. Immunohistochemical (IHC) staining for POSTN, podoplanin (PDPN), and myosin-11 (MYH11) (in brown), on serial sections from two other patient-derived resected pancreatic ductal adenocarcinoma (PDAC) samples showing CAF marker expression in peri-tumoral pancreatitis area. Scale bar, 100 μm.
4. IHC staining for POSTN, PDPN, and MYH11 (in brown) on serial sections from two other KPC mice showing CAF marker expression surrounding acinar-to-ductal metaplasia (ADM) and pancreatic intra-epithelial neoplasia (PanIN) lesions. Scale bar, 200 μm.
5. Multiplex immunofluorescence (IF) co-staining for α-smooth muscle actin (αSMA, in green) and POSTN (in red) showing mutual exclusion (no overlap). Scale bar, 50 μm.
6. Fraction of POSTN-positive cells with (αSMA-positive) or without (αSMA-negative) αSMA co-expression assessed by multiplex IF (quantification of positive pixels, N=9 patients).

**
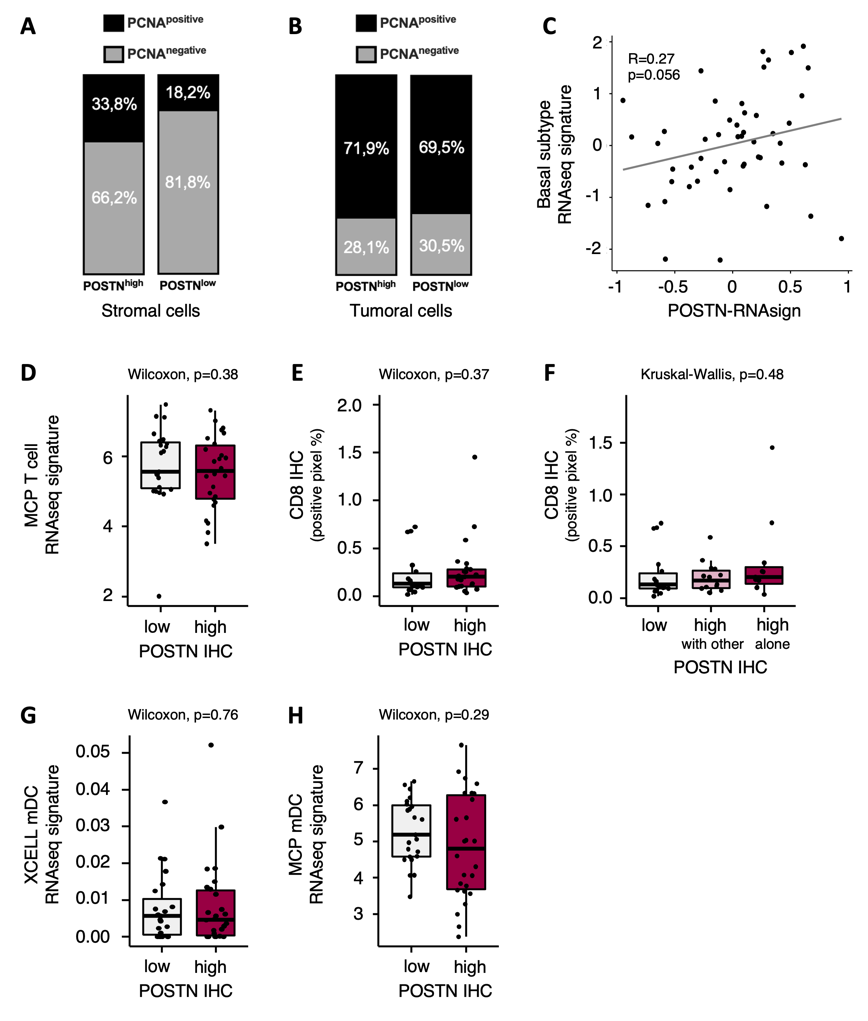
**

**Figure S2. POSTN-high tumors present a higher proliferative stroma and are not associated with T cell or myeloid dendritic cell RNAseq signatures**

1. Fraction of proliferative (proliferating cell nuclear antigen [PCNA]-positive) cells among stromal (pan-cytokeratin [Pan-CK]-negative) nuclei (DAPI) by immunofluorescence staining in periostin (POSTN)-high PDX tumors (N=2, total number of counted nuclei, N=10,630, PCNA-positive N=3,597) versus POSTN-low tumors (N=2, total number of counted nuclei, N=8,162, PCNA-positive N=1,485), classified according to POSTN-RNAsign.
2. Fraction of proliferative (PCNA-positive) cells among tumoral (Pan-CK-positive) nuclei (DAPI) by immunofluorescence staining in POSTN-high PDX tumors (N=2, total number of counted nuclei, N=4,034, PCNA-positive N=2,901) versus POSTN-low tumors (N=2, total number of counted nuclei, N=10,821, PCNA-positive N=7,521), classified according to POSTN-RNAsign.
3. Quantification of basal subtype signature expression level by RNAseq according to POSTN protein expression (high in red versus low in grey) assessed by IHC in resected human PDAC samples (Beaujon cohort, n=50).
4. Quantification of MCP T cell RNAseq signature expression level according to POSTN protein expression (high in red versus low in grey) assessed by IHC in resected human PDAC samples (Beaujon cohort, n=50).
5. Quantification of IHC staining for CD8 (% of positive pixels) according to POSTN protein expression (high in red versus low in grey) assessed by IHC in resected human PDAC samples (Beaujon cohort, n=50).
6. Quantification of IHC staining for CD8 (% of positive pixels) according to POSTN protein expression into 3 classes (POSTN-low in grey, POSTN-high with MYH11 and/or PDPN co-expression in pink, and POSTN-high without MYH11/PDPN co-expression in red) assessed by IHC in resected human PDAC samples (Beaujon cohort, n=50).
7. Quantification of XCELL mDC (myeloid dendritic cells) RNAseq signature expression level according to POSTN protein expression (high in red versus low in grey) assessed by IHC in resected human PDAC samples (Beaujon cohort, n=50).
8. Quantification of MCP mDC RNAseq signature expression level according to POSTN protein expression (high in red versus low in grey) assessed by IHC in resected human PDAC samples (Beaujon cohort, n=50).

**
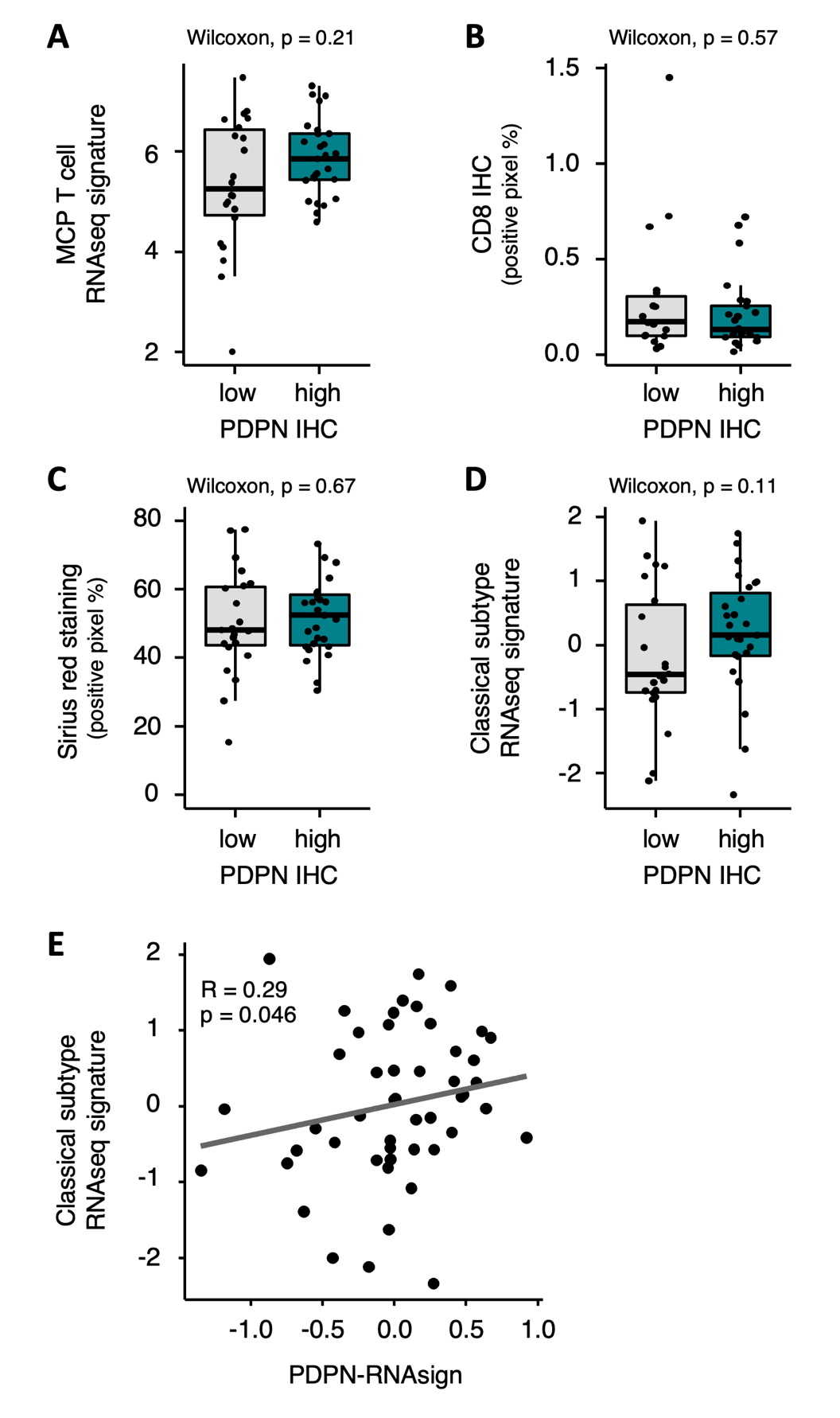
**

**Figure S3.** **PDPN-high tumors are associated with the classical subtype RNAseq signature but not with T cell RNAseq signatures**

1. Quantification of MCP T cell RNAseq signature expression level according to podoplanin (PDPN) protein expression (high in green versus low in grey) assessed by IHC in resected human pancreatic ductal adenocarcinoma (PDAC) samples (Beaujon cohort, n=50).
2. Quantification of IHC staining for CD8 (% of positive pixels) according to PDPN protein expression (high in green versus low in grey) assessed by IHC in resected human PDAC samples (Beaujon cohort, n=50).
3. Quantification of Sirius red stained area (% of positive pixels) according to PDPN protein expression (high in green versus low in grey) assessed by IHC in resected human PDAC samples (Beaujon cohort, n=50).
4. Quantification of classical subtype signature expression level by RNAseq according to PDPN protein expression (high in green versus low in grey) assessed by IHC in resected human PDAC samples (Beaujon cohort, n=50).
5. Correlation between PDPN multigene RNAseq signature (PDPN-RNAsign) expression and classical subtype signature expression by RNAseq in resected human PDAC samples (Beaujon cohort, n=50).

**
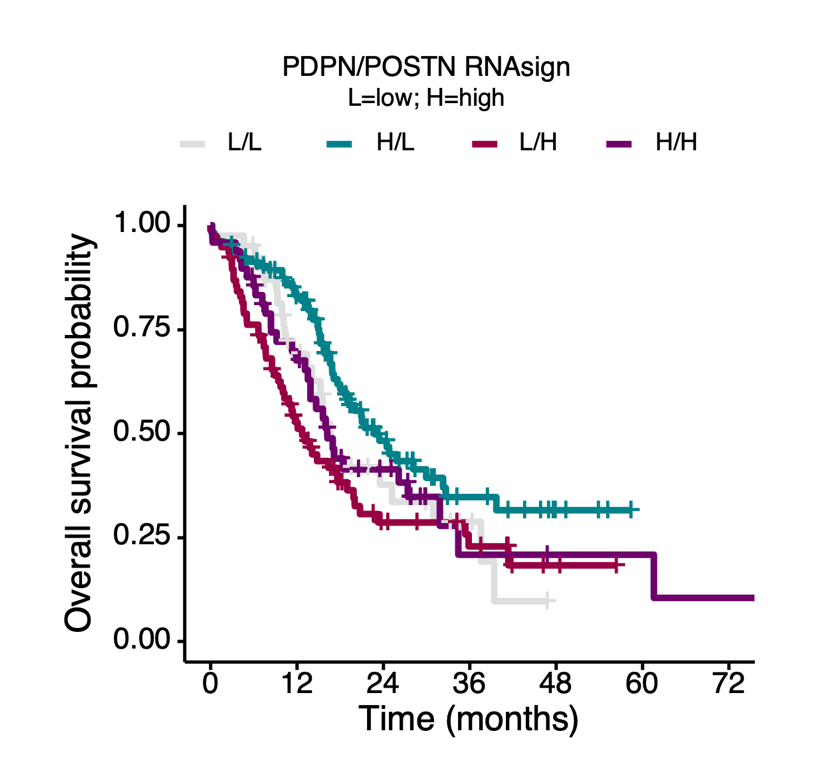
**

**Figure S4. Kaplan–Meier curves for overall survival (OS) in the ICGC cohort (n=247), according to combined POSTN and PDPN multigene RNAseq signatures**

**
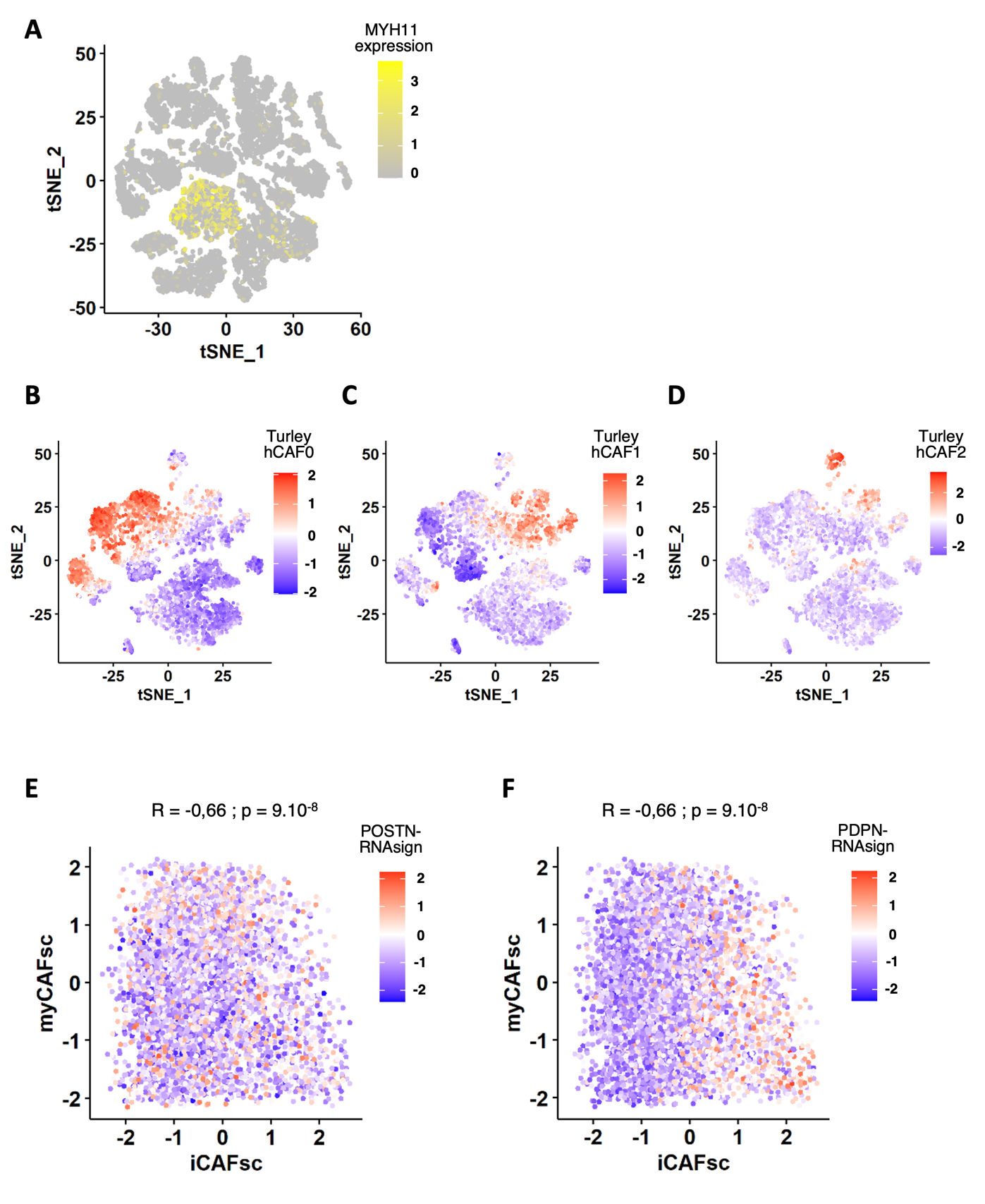
**

**Figure S5. CAF single cell analyses**

1. Plot of expression levels of myosin-11 (MYH11) in each analyzed cell (all cell types) onto the t-SNE map. Color key from grey to yellow indicates relative expression levels from low to high.
2. Plot of hCAF0 (Turley’s group [15]) RNAseq signature expression level in each analyzed cell (fibroblasts only). Higher expression is shown in red and lower expression in blue.
3. Plot of hCAF1 (Turley’s group [15]) RNAseq signature expression level in each analyzed cell (fibroblasts only). Higher expression is shown in red and lower expression in blue.
4. Plot of hCAF2 (Turley’s group [15]) RNAseq signature expression level in each analyzed cell (fibroblasts only). Higher expression is shown in red and lower expression in blue.
5. Plot of fibroblasts according to myofibroblastic CAF (myCAF, Tuveson’s group [12]) and inflammatory CAF (iCAF, Tuveson’s group [12]) RNAseq signatures, and POSTN multigene RNAseq signature (POSTN-RNAsign) expression level. Higher expression is shown in red and lower expression in blue.
6. Plot of fibroblasts according to myCAF and iCAF (Tuveson’s group [12]) RNAseq signatures, and PDPN multigene RNAseq signature (PDPN-RNAsign) expression level. Higher expression is shown in red and lower expression in blue.
