# Supplementary material for: Periostin‐ and podoplanin‐positive cancer‐associated fibroblast subtypes cooperate to shape the inflamed tumor microenvironment in aggressive pancreatic adenocarcinoma
Source: J Pathol. 2022 Oct 21;258(4):408–25. doi: 10.1002/path.6011 (PMC9828775; doi:10.1002/path.6011)
Supplement: Supplementary file 3 — Table S2. Distribution of samples from the five data sets according to periostin and podoplanin expression (from RNAseq signatures) Table S3. Antibodies used for immunohistochemistry on resected pancreatic ductal adenocarcinoma formalin‐fixed and paraffin‐embedded samples from Beaujon patient cohort and from KPC models Table S4. Antibodies used for multiplex immunofluorescence Table S5. Transcripts used to define cell type clusters for single‐cell analyses [file PATH-258-408-s001.docx]

**Periostin- and podoplanin- positive cancer-associated fibroblast subtypes cooperate to shape the inflamed tumor microenvironment in aggressive pancreatic adenocarcinoma**

C Neuzillet *et al. J Pathol* DOI: <https://doi.org/10.1002/path.6011>

**Supplementary Table S1 (separate Excel file)**

**Supplementary Tables S2–S5**

**Table S2.** **Distribution of samples from the five datasets according to periostin (POSTN) and podoplanin (PDPN) expression (from RNAseq signatures)**

|  | POSTN – N (%) | | PDPN – N (%) | |
| --- | --- | --- | --- | --- |
| Dataset | POSTN – | POSTN + | PDPN – | PDPN + |
| TCGA (N=150)  Moffitt (N=206)  ICGC (N=269)  Puleo (N=309)  PDX stroma (N=90) | 81 (54.0%)  90 (43.7%)  131 (48.7%)  143 (46.3%)  48 (53.3%) | 69 (46.0%)  116 (56.3%)  138 (51.3%)  166 (53.7%)  42 (46.7%) | 64 (42.7%)  93 (45.1%)  114 (42.4%)  147 (47.6%)  50 (55.6%) | 86 (57.3%)  113 (54.9%)  155 (57.6%)  162 (52.4%)  40 (44.4%) |
| 𝜒^2^ test, p | 0.2740 | | 0.2197 | |

**Table S3.** **Antibodies used for immunohistochemistry on resected PDAC FFPE samples from the Beaujon patient cohort and from KPC models**

| **Primary**  **antibody to** | **Species**  **raised in** | **Supplier**  **(catalogue no.)** | **Dilution** | **Detection** |
| --- | --- | --- | --- | --- |
| **Periostin (POSTN)** | Rabbit | Abcam, Cambridge, UK  (ab219057) | 1:500 | iVIEW DAB Detection Kit, Ventana, Roche |
| **Myosin 11 (MYH11)** | Rabbit | Novus Biological, Centennial, CO, USA (NB600-1032) | 1:200 | iVIEW DAB Detection Kit, Ventana, Roche |
| **Podoplanin (PDPN)** | Rabbit | Sigma-Aldrich, St. Louis, MO, USA (HPA007534) | 1:250 | iVIEW DAB Detection Kit, Ventana, Roche |
| **αSMA** | Mouse | Dako, Glostrup, Denmark  (M0851) | 1:500 | iVIEW DAB Detection Kit, Ventana, Roche |
| **Pan-CK** | Mouse | Zytomed System, Berlin, Germany  (MSK098-05) | 1:200 | iVIEW DAB Detection Kit, Ventana, Roche |
| **CD163** | Mouse | Leica Biosystems, Nussloch, Germany  (NCL-CD163) | 1:100 | iVIEW DAB Detection Kit, Ventana, Roche |
| **CD8** | Mouse | Dako  (M710301) | 1:50 | iVIEW DAB Detection Kit, Ventana, Roche |

αSMA: α-smooth muscle actin, Pan-CK: pan-cytokeratin.

**Table S4.** **Antibodies used for multiplex immunofluorescence**

| **Antibody against** | **Species raised in** | **Supplier (catalogue no.)** | **Dilution** |
| --- | --- | --- | --- |
| *Human tumors* | | | |
| αSMA | Mouse | M0851, Dako, Glostrup, Denmark | 1/600 |
| Pan-CK | Mouse | MSKO98-05, Zytomed Systems, Berlin, Germany | 1/200 |
| POSTN | Rabbit | ab219057, Abcam, Cambridge, UK | 1/500 |
| Opal® 4-Color Automation IHC kit | N/A | Akoya Biosciences, Menlo Park, CA, USA | N/A |
| *Patient-derived xenografts* | | | |
| PCNA | Mouse | PC-10, sc-56, Santa Cruz Biotechnology, Dallas, TX, USA | 1/300 |
| Pan-CK (cocktail) Alexa Fluor 647 | Mouse | NBP2-7642AF647, Novus Biologicals, Centennial, CO, USA | 1/100 |
| POSTN | Rabbit | H-300, sc-67233, Santa Cruz Biotechnology | 1/100 |
| αSMA | Mouse | ab7817, Abcam | 1/200 |
| Anti-rabbit IgG Alexa Fluor 488 | Goat | A27034, Invitrogen, Waltham, MA, USA | 1/1,000 |
| Anti-mouse IgG Alexa Fluor 546 | Goat | A11030, Invitrogen | 1/1,000 |
| DAPI | N/A | #D9545, Sigma-Aldrich, St. Louis, MO, USA | 1/1,000 |

αSMA: α-smooth muscle actin, Pan-CK: pan cytokeratin, PCNA: proliferating cell nuclear antigen, POSTN: periostin, N/A: not applicable.

**Table S5. Transcripts used to define the cell type clusters for single-cell analyses**

| **Cell types** | **Gene Symbol** |
| --- | --- |
| **B lineage** | *BANK1, CD19, CD22, CD79A, CR2, FCRL2, IGKC, MS4A1, PAX5* |
| **CD8 T cells** | *CD8B* |
| **Cytotoxic lymphocytes** | *CD8A, EOMES, FGFBP2, GNLY, KLRC3, KLRC4, KLRD1* |
| **Endothelial cells** | *ACVRL1, APLN, BCL6B, BMP6, BMX, CDH5, CLEC14A, CXorf36, EDN1, ELTD1, EMCN, ESAM, ESM1, FAM124B, HECW2, HHIP, KDR, MMRN1, MMRN2, MYCT1, PALMD, PEAR1, PGF, PLXNA2, PTPRB, ROBO4, SDPR, SHANK3, SHE, TEK, TIE1, VEPH1, VWF* |
| **Epithelial** | *KRT19, CDH1, MUC1, SOX9, EPCAM* |
| **Fibroblasts** | *COL1A1, COL3A1, COL6A1, COL6A2, DCN, GREM1, PAMR1, TAGLN* |
| **Monocytic lineage** | *ADAP2, CSF1R, FPR3, KYNU, PLA2G7, RASSF4, TFEC* |
| **Myeloid dendritic cells** | *CD1A, CD1B, CD1E, CLEC10A, CLIC2, WFDC21P* |
| **Neutrophils** | *CA4, CEACAM3, CXCR1, CXCR2, CYP4F3, FCGR3B, HAL, KCNJ15, MEGF9, SLC25A37, STEAP4, TECPR2, TLE3, TNFRSF10C, VNN3* |
| **NK cells** | *CD160, KIR2DL1, KIR2DL3, KIR2DL4, KIR3DL1, KIR3DS1, NCR1, PTGDR, SH2D1B* |
| **T cells** | *CD28, CD3D, CD3G, CD5, CD6, CHRM3-AS2, CTLA4, FLT3LG, ICOS, MAL, MGC40069, PBX4, SIRPG, THEMIS, TNFRSF25, TRAT1* |
